# Quantitative Analysis of the Epoxidation of Waste Cooking Oil Biodiesel by ^1^H NMR

**DOI:** 10.1021/acsomega.5c11222

**Published:** 2026-07-06

**Authors:** João F. A. Costa, Leiliane do S. S. de Souza, Kidney O. G. Neves, Marcos B. Machado, Luiz K. C. de Souza, Sergio D. Junior, Eduardo A. C. Batista, Ramon S. B. Ferreira, Anderson M. Pereira, João N. N. Quaresma

**Affiliations:** † Graduate Program in Engineering of Natural Resources of the Amazon (PRODERNA), Institute of Technology, Federal University of Pará (UFPA), Belém, Pará 66075-110, Brazil; ‡ Department of Agricultural and Soil Engineering, Faculty of Agricultural Sciences, Federal University of Amazonas (UFAM), Manaus, Amazonas 69080-900, Brazil; § Nuclear Magnetic Resonance Laboratory (NMRLAB), Analytical Center, Multidisciplinary Support Center, Federal University of Amazonas (UFAM), Manaus, Amazonas 69080-900, Brazil; ∥ Fuel Research and Testing Laboratory (LAPEC), Department of Chemistry, Institute of Exact Sciences, Federal University of Amazonas (UFAM), Manaus, Amazonas 69080-900, Brazil; ⊥ Chemical Analysis Center (CAQQAT), School of Technology, Amazonas State University (UEA), Manaus, Amazonas 69050-020, Brazil; # Extraction, Applied Thermodynamics, and Equilibrium Laboratory (EXTRAE), School of Food Engineering, University of Campinas (UNICAMP), Campinas, São Paulo 13083-862, Brazil; ∇ Center for Social Sciences, Health, and Technology, Federal University of Maranhão (UFMA), Imperatriz, Maranhão 65915-240, Brazil

## Abstract

The valorization of waste cooking oils (WCO) through biodiesel production represents a sustainable strategy aligned with circular-economy principles. Beyond its use as a renewable fuel, biodiesel serves as a precursor of high-value derivatives such as biolubricants and polymer additives. In this study, methyl (MB) and ethyl (EB) esters derived from WCO biodiesel were epoxidized using peracetic acid. Quantitative ^1^H NMR (q ^1^H NMR) spectroscopy was applied, using the terminal methyl group as an internal reference and the oxirane protons for quantification. Maximum conversions of 74.25 ± 0.04% (MB) and 80.62 ± 0.11% (EB) were achieved at 1.67% and 2.00% peracetic acid, respectively. Higher concentrations led to decreased conversions due to oxirane-ring opening and secondary oxidation. Ethyl esters showed higher reactivity but also a greater degradation tendency. The developed q ^1^H NMR method proved robust and industrially relevant, establishing ^1^H NMR as a reliable quantitative tool for monitoring biodiesel epoxidation reactions.

## Graphical abstract

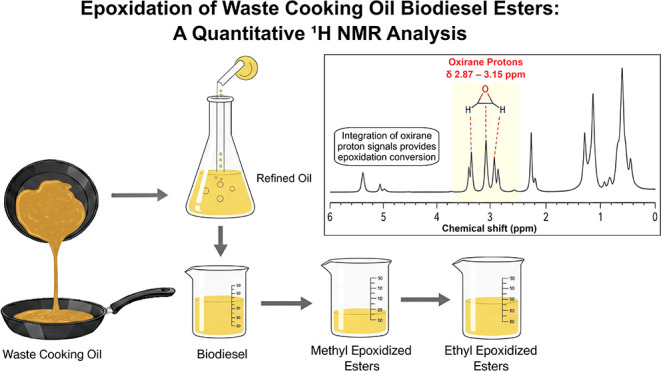

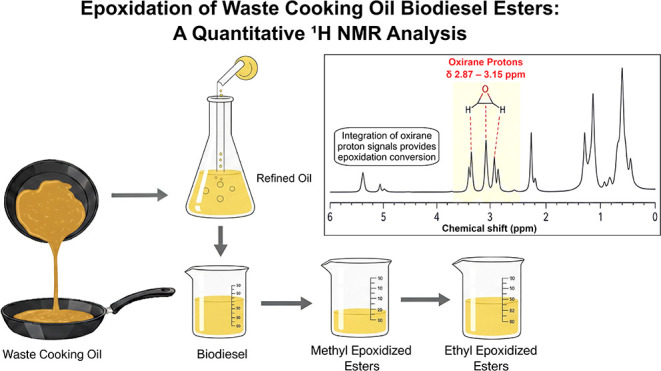

## 1 Introduction

The search for sustainable energy solutions has driven the reuse of oily residues for the production of biofuels and high value-added inputs. The valorization of waste cooking oils (WCO) stands out as a strategic alternative, as it reduces environmental impacts and enables the generation of renewable-based chemical products.^1–3^ Among these, biodiesel occupies a prominent position due to its properties such as biodegradability, low toxicity, and reduced emissions of regulated pollutants in exhaust gases (CO, particulate matter, and hydrocarbons), which are well documented for WCO-derived biodiesel.^4^ In addition, life cycle assessment studies report a net reduction in regulated pollutants when biodiesel produced from oily waste streams is employed.^5^

Recent studies further reinforce that the use of WCO directly contributes to the circular economy and to the mitigation of impacts associated with the improper disposal of lipid residues, expanding its potential application in sustainable chemical routes.^1,2,6^

The use of WCO as a feedstock for epoxidation has been identified as a highly promising alternative for converting waste into higher value-added products, particularly when combined with other lipid sources in hybrid systems.^7^

Beyond its role as a fuel, biodiesel constitutes a versatile platform for the synthesis of industrial derivatives, such as biolubricants and plasticizers obtained from epoxidized esters, which exhibit superior thermal and oxidative stability and technical performance comparable to petrochemical analogues in terms of viscosity, volatility, and shear stability.^8–10^ Such compounds show enhanced oxidative stability and performance similar to petrochemical products, reinforcing the principles of green chemistry and the circular economy.^10^

Recent reviews demonstrate that epoxides derived from fatty acids exhibit excellent performance as polymer additives, plasticizers, and functional fluids, strengthening their potential for applications as biolubricants and in PVC formulations and composite materials.^10^

Among derivative processes, the epoxidation of unsaturated esters represents a promising chemical route. The introduction of oxirane groups into fatty chains increases viscosity and improves the thermal and oxidative stability of fatty esters, as reported in studies on the properties of epoxidized vegetable oils.^11^ These materials also exhibit potential for application as polymer additives and plasticizers.^12^ The efficiency of epoxidation strongly depends on reaction conditions and on the oxidizing agent employed. This behavior has also been observed in recent studies on the epoxidation of WCO mixtures and fatty acids, in which temperature, agitation, and molar ratio significantly influenced oxirane yield.^7^

Although epoxidation and transesterification processes employ homogeneous catalysts, which may initially appear contrary to green chemistry principles, their use remains widely documented due to high catalytic activity, operational simplicity, and low cost. In systems derived from waste oils, after proper neutralization of acidity and control of moisture, these catalysts exhibit high efficiency and good tolerance to feedstock compositional variability, justifying their application in both industrial and academic contexts.^3,13,14^ Peracetic acid, due to its high selectivity and lower toxicity, has been widely applied as a substitute for performic and perbenzoic acids.^4,15^

Recent studies indicate that the use of peracetic acid formed *in situ* allows WCO epoxidation to be conducted under mild conditions, reducing oxirane ring opening and the formation of byproducts, while also offering greater operational safety and improved thermal stability of the oxidant.^3,7,13,16^

To evaluate the extent of these transformations, proton nuclear magnetic resonance spectroscopy (^1^H NMR) has emerged as a robust, selective, and quantitative analytical tool, enabling the correlation of spectral integrals with chemical conversion.^17,18^ The application of quantitative NMR (q ^1^H NMR) allows rapid and accurate determination of the quantitative chemical composition of complex organic matrices without the need for chromatographic methods,^19^ as well as quantification of the fraction of double bonds converted into oxirane groups, eliminating the need for complex chromatographic steps.^17^

However, for complex matrices such as waste oils, which contain mixtures of fatty acids with different degrees of unsaturation, NMR-based quantification requires special attention to avoid spectral overlap interference, ensuring proper validation of integration windows and experimental parameters.^20–22^

Therefore, the main objective of the present study was to investigate the epoxidation process of methyl (MB) and ethyl (EB) esters derived from waste-oil biodiesel, using peracetic acid as the epoxidizing agent and quantitative ^1^H NMR for determination of the conversions achieved. In doing so, this work aims to contribute to the development of biolubricants and sustainable inputs of industrial interest.

Additionally, the influence of peracetic acid concentration on reaction selectivity was evaluated, as well as the differences in behavior between MB and EB, providing a comparative analysis capable of supporting future optimization strategies and industrial applications of these materials.^23,24^

## 2 Experimental Section

### 2.1 Reagents

Waste frying oils were collected from restaurants in Manaus (AM, Brazil). Methanol (P.A., ACS/ISO grade) and anhydrous ethanol (DINÂMICA), potassium hydroxide (KOH, 85%, DINÂMICA), acetic anhydride (C_4_H_6_O_3_, CROMOLINE), 50% hydrogen peroxide (DINÂMICA), and commercial 15% peracetic acid (PROXITANE 1512) were used. Deuterated chloroform (CDCl_3_), used to prepare the samples for NMR analysis, was obtained from Cambridge Isotope Laboratories Inc. (Andover, MA, USA). Dimethyl terephthalate (DMT), a Certified Reference Material (CRM), was issued by the Chemical and Thermal Metrology Division (Inmetro, Rio de Janeiro, Brazil) under certificate number DIMCI1507/2019 (certified purity: 999.88 ± 0.060%).

### 2.2 Waste Cooking Oil (WCO)

The waste cooking oil (WCO) was vacuum-filtered using medium-porosity filter paper (14 μm) to remove suspended solids and subsequently subjected to acid value and water content analyses according to the official AOCS methods Cd 3d-63 (2017) and Ca 2e-84 (2009).^25^ The refining step followed the procedures described by Gharby et al.^26^ The values obtained were compared with the limits recommended by Gerpen^27^ for alkaline transesterification reactions.

### 2.3 Production of Methyl (MB) and Ethyl (EB) Esters

Figure 1 presents the reaction scheme of the transesterification process employed in this study, describing the conversion of triacylglycerides present in waste cooking oil into fatty acid esters (biodiesel) and glycerol. The experimental conditions used for the production of methyl and ethyl esters are detailed below. Transesterification reactions were carried out using an oil-to-alcohol molar ratio of 1:10, temperature of 60 °C, and KOH at 2.0 wt % as catalyst. These conditions are consistent with optimization studies employing Taguchi methodology and ANOVA, which demonstrated yields higher than 96% under similar parameters,^28^ as well as with studies reporting 91.6% conversion using KOH at moderate temperatures.^29^ Conventional heating was employed, which has shown results comparable to microwave heating in ANOVA-optimized studies,^30^ with the advantage of greater operational simplicity and scalability. After 1 h of reaction, excess alcohol was removed by evaporation. The product was washed with distilled water until neutral pH, centrifuged (3500 rpm), and dried with anhydrous sodium sulfate. Subsequently, the salt was removed by filtration, and the biodiesel was stored in amber bottles at approximately 20 °C for further analyses. Physicochemical characterization included acid value and water content according to AOCS methods Cd 3d-63 (2017) and Ca 2e-84 (2009),^25^ density measured directly using a DMA 4101 densimeter (Anton Paar, Austria), and viscosity determined according to ASTM D445 (2019).^31^

**Figure 1:**
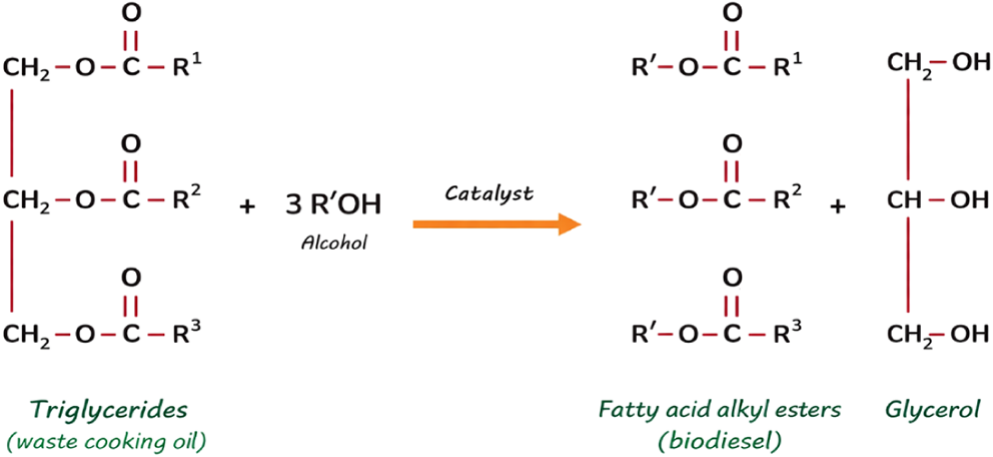
Reaction scheme of the transesterification of triglycerides with alcohol to produce fatty acid esters (biodiesel) and glycerol.

### 2.4 Production of Peracetic Acid

Peracetic acid was synthesized in the laboratory by reaction between acetic anhydride and 50% hydrogen peroxide at mass ratios of 1:1, 3:1, 3:1.5, 3:2, and 4.5:1 (C_4_H_6_O_3_/H_2_O_2_). This synthesis route allows *in situ* formation of the oxidizing agent, avoiding stability and transportation issues associated with commercial peracetic acid,^32^ and has been widely employed for the epoxidation of vegetable oils and derivatives. Variation of the mass ratios enables evaluation of the effect of oxidant concentration on double-bond conversion, a critical parameter for maximizing the degree of epoxidation, as demonstrated in systematic studies.^33^ Acetic anhydride was maintained under vigorous stirring in an ice bath while hydrogen peroxide was added dropwise. The reaction mixture was maintained for 4 h at 40 ± 5 °C and stored in a refrigerated amber bottle. The peracetic acid concentration was determined by titration according to the methodology described by Evonik Operations GmbH.^34^

The formation of peracetic acid and its kinetic behavior follow well-established partition models,^35^ which describe the distribution of the oxidant between aqueous and organic phases during the subsequent epoxidation step.

Accordingly, peracetic acid was obtained by reaction between acetic anhydride and hydrogen peroxide, as illustrated in Figure 2.

**Figure 2:**
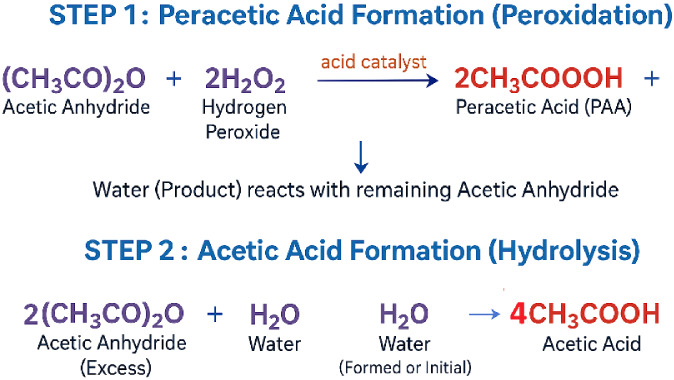
Overall reaction pathway for the synthesis of PAA from acetic anhydride, highlighting the subsequent formation of acetic acid via water-mediated hydrolysis.

### 2.5 Epoxidation Reactions

MB and EB esters were epoxidized using different peracetic acid concentrations (1.65%, 1.67%, 2.00%, 2.08%, 2.16%, and 15.00%) at 45 ± 5 °C under moderate stirring (250 rpm) for 1 h. Thus, six epoxidized products were obtained for each biodiesel, resulting in a total of 12 epoxides (six from MB and six from EB). The reactions were conducted using equimolar proportions (1:1) of ester and peracetic acid. After reaction, the phases were separated using a separatory funnel: an epoxide-rich phase (upper phase) and an acetic-acid-rich phase (lower phase). After removal of the lower phase, the epoxides were washed three times with 100 mL of 10% NaHCO_3_ solution until complete cessation of gas evolution due to the neutralization reaction and then centrifuged at 3500 rpm for 15 min. The product was subsequently dried with anhydrous magnesium sulfate to remove residual water. Magnesium sulfate was later removed by vacuum filtration. The epoxides were stored in refrigerated amber bottles until analysis. Figure 3 presents the reaction scheme of the epoxidation employed in this study, highlighting the conversion of C=C bonds of methyl and ethyl esters into oxirane groups.

**Figure 3:**
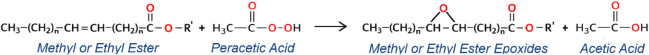
General reaction scheme for the epoxidation of methyl or ethyl esters using peracetic acid, illustrating the conversion of C=C double bonds into oxirane rings.

### 2.6 ^1^H NMR Analysis

NMR analyses were performed on a Bruker Avance III HD NMR spectrometer (Bruker, MA, USA), operating at 11.7 T (500 MHz for ^1^H) and equipped with a 5 mm BBFO Plus SmartProbe with a *Z*-axis gradient. Approximately 15 mg (in triplicate) of each ester and epoxide were dissolved in 550 μL of CDCl_3_ containing 3.09 mM DMT (dimethyl terephthalate) as an internal standard for quantification. ^1^H NMR spectra were acquired at 25 °C using the *zg* pulse sequence. The 90° pulse length (P1 = 8.00 μs) and relaxation delay (d1 = 21.97 s) were determined for the signal at δ 3.96 (6H). A total of 2 dummy scans and 8 scans were collected with 64k data points, using a spectral width of 10 kHz and an acquisition time of 3.27 s; the receiver gain was set to 32. Phase and baseline corrections were performed manually using TopSpin 3.6.3 software.

#### 2.6.1 Quantification of MB and EB by ^1^H NMR

The conversion of waste frying oil into methyl biodiesel (MB) and ethyl biodiesel (EB) was determined by ^1^H NMR using the internal standard method for quantification. Spectra were acquired under conditions ensuring complete relaxation of the analyzed hydrogen nuclei, using dimethyl terephthalate (DMT, 3.09 mM) as a certified internal standard, thereby ensuring metrological traceability and quantitative comparability among samples.^19^

Although the relative quantification of the spectral signals was ensured by the use of an internal standard, biodiesel conversion was expressed as functional ratios between characteristic signals of the system itself, following methodologies widely established in the literature for the spectroscopic determination of fatty acid ester yields.^36,37^ This approach enables assessment of transesterification efficiency without assuming absolute structural conversion of triacylglycerols.

For methyl biodiesel (MB), conversion was calculated from the ratio between the integral of the methoxy proton signal (−OCH_3_), observed at approximately δ 3.65, and the integral of the terminal methyl signal (ω-CH_3_), centered at approximately δ 0.88, according to eq 1:^36^

1 $$C_{\mathrm{MB}\;}=\frac{I_{{\mathrm{OCH}}_{3}}}{I_{\omega {\mathrm{‐CH}}_{3}}}\times 100\%$$

For ethyl biodiesel (EB), conversion was determined from the ratio between the integral of the ethoxy methylene proton signal (−CH_2_–O−), observed in the δ 4.05–4.20, and the integral of the terminal methyl signal (ω-CH_3_) in δ 0.88, applying the stoichiometric correction factor corresponding to the number of protons involved, as described in eq 2:^37^

2 $$C_{\mathrm{EB}\;}=\frac{3}{2}\times \frac{I_{{\mathrm{OCH}}_{2}}}{I_{\omega {\mathrm{‐CH}}_{3}}}\times 100\%$$

The terms used in eqs 1 and 2 are described in Table 1.

**Table 1:** Terms, Definitions, and Spectral Regions Used for ^1^H NMR Quantification of Methyl (MB) and Ethyl (EB) Biodiesel

symbol	meaning	region (δ, ppm)	note
*I*_OCH_3__	integral of methoxyl protons (−OCH_3_) of MB	3.64–3.68	direct evidence of methyl ester formation
*I*_ω‑CH_3__	integral of terminal methyl protons of fatty chain	0.80–0.90	internal reference is present in all esters
*I*_OCH_2__	integral of ethoxyl protons (−CH_2_–O−) of EB	4.06–4.18	indicator of ethyl ester formation
3/2	stoichiometric correction factor	–	adjusts OCH_2_ (2 H) vs CH_3_ (3 H) in ratio
*C*_MB_, *C*_EB_	percent conversion of the respective biodiesel	–	spectroscopic yield of esters formed

It is emphasized that, although eqs 1 and 2 are expressed as integral ratios, the values used were obtained from the areas of the signals of interest normalized to the dimethyl terephthalate signal at δ 3.96 (6 H), which provides greater quantitative robustness to the calculated conversions. Thus, the results ensure methodological consistency between the quantification of biodiesel (MB and EB) and the quantification of the epoxidized products described in Section 2.6.2. The conversions obtained therefore represent functional spectroscopic yields of fatty acid ester formation, suitable for comparing the methyl and ethyl routes and consistent with the analytical limitations inherent to ^1^H NMR.

#### 2.6.2 Quantification of Epoxides by ^1^H NMR

The conversion of double bonds into oxirane groups was determined by quantitative ^1^H NMR, following methodologies described by Aerts and Jacobs^38^ and adapted for methyl and ethyl fatty acid esters by Xia et al.^18^ This conversion of double bonds into oxirane rings was quantified using a stoichiometric ^1^H NMR approach, based on the decrease in the area of olefinic protons and the concomitant formation of oxirane protons, normalized to the initial DB_0_ value (initial average number of double bonds per chain). Although different analytical techniques have been proposed for epoxide determination, this NMR-based method has proven reliable for monitoring epoxidation reactions of fatty acid esters.

The spectral regions selected for integration were δ 2.87–3.15 (oxirane protons, CH–O–CH), δ 5.28–5.56 (olefinic protons, CH=CH), and δ 0.80–0.90 (terminal methyl protons, ω–CH_3_). The choice of these regions was based on the absence of overlap with signals from other components of the system.^18,39^ The terminal methyl group (ω–CH_3_) was used as an invariant spectroscopic signal for internal normalization, whereas absolute quantification was ensured by using a certified internal standard (DMT), as described in Section 2.6. This choice was made because the ω–CH_3_ group is present in all chains and remains unchanged during epoxidation, a strategy that is well established in NMR-based transesterification studies.^36,40^ The initial average number of double bonds per chain (DB_0_) was determined from the starting biodiesel by the ratio between the mean integrated areas of the olefinic and terminal methyl signals.

In this work, DB_0_ is defined as the initial average number of double bonds per fatty acyl chain, determined experimentally by quantitative ^1^H NMR from the starting biodiesel, constituting a functional mean metric that is valid for polyunsaturated mixtures. Accordingly, the following equations were formulated to provide average estimates of the functional conversion of chemical groups detectable by ^1^H NMR, rather than to describe the absolute structural conversion of individual molecular species in polyunsaturated mixtures. Thus, eq 3 was used to determine DB_0_:

3 $${\mathrm{DB}}_{0}=\frac{{\mathrm{I̅}}_{\mathrm{olefinic}}}{{\mathrm{I̅}}_{{\mathrm{CH}}_{3}}}\times \frac{3}{2}$$

where *I̅*_olefinic_ corresponds to the mean integrated area of the olefinic protons (δ 5.28–5.56) and *I̅*_CH_3__ to the mean integrated area of the terminal methyl protons (δ 0.80–0.90). The (3/2) factor corrects for the stoichiometric difference between the three protons of the CH_3_ group and the two protons associated with the double bond.^36^ In this context, DB_0_ represents the initial average number of double bonds per fatty acyl chain, determined experimentally by ^1^H NMR from the ratio between olefinic and terminal (ω–CH_3_) protons, constituting a functional mean metric that is valid for complex mixtures of fatty acid esters. The average conversion of double bonds into oxirane groups (*C*) was then calculated using eq 4.

4 $$C=\frac{I_{\mathrm{epoxide}}}{I_{{\mathrm{CH}}_{3}}}\times \frac{3}{2}\times \frac{1}{{\mathrm{DB}}_{0}}\times 100\%$$

where *I*_epoxide_ is the integrated area of the oxirane protons (δ 2.87–3.15). This relationship expresses the fraction of the initial unsaturations (DB_0_) effectively converted into oxiranes, determined by quantitative NMR and normalized to an invariant signal of the fatty acyl chain. The use of this formulation enables quantitative and reproducible comparison of the epoxidation series of methyl (EBM) and ethyl (EBE) biodiesels, minimizing the influence of sample mass and instrumental gain.^18^

In systems containing polyunsaturated fatty acid esters, eq 4 provides the overall average conversion of double bonds into oxirane groups detectable by NMR, without distinguishing between mono-, di-, or poly epoxidation of individual chains. The terms used in eqs 3 and 4 are described in Table 2.

**Table 2:** Terms, Definitions, and Spectral Regions Used for ^1^H NMR Quantification of Epoxidized Methyl (MB) and Ethyl (EB) Fatty Acid Esters

symbol	meaning	region (δ, ppm)	note
*I*_epoxide_	integral of formed oxirane protons (−CH–O–CH−)	2.87–3.15	proportional to the number of oxiranes formed
*I*_CH_3__	integral of terminal methyl protons (ω–CH_3_)	0.80–0.90	stable internal reference
*I̅*_olefinic_	mean integrated area of olefinic protons (CH=CH) before the reaction	5.28–5.56	determines the initial DB_0_ of the substrate
DB_0_	initial average number of double bonds per chain	–	obtained from eq 3 on starting biodiese
3/2	stoichiometric correction factor between protons	–	adjusts CH_3_ (3 H) vs CH=CH (2 H)
*C*	fraction of double bonds converted into oxiranes	–	spectroscopic yield of epoxidation

## 3 Results and Discussion

### 3.1 Characterization of Waste Cooking Oil (WCO)

The waste cooking oil (WCO) presented an initial acid value of 4.48 ± 0.01 mg KOH g^–1^, corresponding to 2.25% free fatty acids, which is above the limit recommended for alkaline transesterification (≤2.0 mg KOH g^–1^).^27^ This behavior is common in reused oils, in which thermal degradation promotes partial hydrolysis of triacylglycerides and the formation of free fatty acids. After neutralization, the acid value was reduced to 0.25 ± 0.04 mg KOH g^–1^, corresponding to 0.12% free fatty acids, demonstrating the efficiency of the pretreatment step. The moisture content was 0.05 ± 0.02 mg kg^–1^, at the maximum allowable limit, which requires strict control to avoid emulsion formation and hydrolysis during transesterification.^41^

### 3.2 Characterization of Methyl (MB) and Ethyl (EB) Biodiesel

As summarized in Tables 3 and 4, the physicochemical properties of methyl (MB) and ethyl (EB) biodiesel produced under an oil-to-alcohol molar ratio of 1:10 at 60 °C and using 2.0 wt % KOH, are close to the limits established by ANP Resolution No. 920/2023 ^42^, confirming the adequacy of the transesterification conditions adopted in this study.

**Table 3:** Physicochemical Properties of Methyl (MB) and Ethyl (EB) Biodiesel Derived from Waste Cooking Oil

property	unit	MB	EB	notes
acid value	mg KOH g^–1^	0.42	0.53	AOCS Cd 3d-63
water content	mg kg^–1^	190.0 ± 0.12	208.0 ± 0.31	AOCS Ca 2e-84
density (20 °C)	kg m^–3^	882	893	DMA 4101
kinematic viscosity (40 °C)	mm^2^ s^–1^	5.80 mm^2^ s^–1^	6.20	ASTM D445

**Table 4:** Compliance of MB and EB with ANP Resolution No. 920/2023 Specifications

parameter	ANP limit	MB	EB	compliance
acid value (mg KOH g^–1^)	≤0.50	0.42	0.53	MB: yes/EB: slight exceedance
water content (mg kg^–1^)	≤200	190.0 ± 0.12	208.0 ± 0.31	MB: yes/EB: no
kinematic viscosity (40 °C)	3.0–6.0	5.80	6.20	MB: yes/EB: no
density (20 °C)	850–900	882	893	Both: yes

Regarding acidity, MB exhibited an acid value of 0.42 mg KOH g^–1^, while EB presented a slightly higher value of 0.53 mg KOH g^–1^ (Table 3). Although the ethyl route shows a marginal increase, both values are within acceptable limits, indicating effective neutralization and washing steps. This behavior is consistent with the known higher sensitivity of ethyl transesterification systems to residual free fatty acids and moisture when compared to the methyl route.

The water content, reported in Table 3, highlights a clear distinction between the two biodiesel types. MB showed a moisture content of 190.0 ± 0.12 mg kg^–1^, complying with the maximum limit of 200 mg kg^–1^ established by ANP Resolution No. 920/2023,^42^ whereas EB presented a slightly higher value of 208.0 ± 0.31 mg kg^–1^, exceeding the regulatory threshold. This result is consistent with the higher hygroscopicity of ethyl esters. Moisture control is particularly critical, since elevated water contents during alkaline transesterification promote saponification and emulsion formation, leading to reduced ester yield.^41^

From the perspective of downstream processing, the higher moisture content of EB may also affect subsequent acidic epoxidation reactions. As discussed in the manuscript, residual water does not generate soaps under acidic conditions but may favor oxirane ring opening reactions, forming diols and consuming part of the peracetic acid, thereby reducing reaction selectivity.^11,43^ Thus, the values reported in Table 3 anticipate potential differences in epoxidation behavior between MB and EB.

The kinematic viscosity at 40 °C further differentiates the two biodiesels. EB exhibited a viscosity of 6.20 mm^2^ s^–1^, slightly above the upper limit of 6.0 mm^2^ s^–1^ specified by ANP Resolution No. 920/2023 ^42^, whereas MB remained within the regulatory range (Table 3). This increase in viscosity is attributed to the higher molar mass and lower volatility of ethyl esters. In parallel, EB also presented a higher density (893 kg m^–3^) than MB (882 kg m^–3^), a factor that may influence oxidant diffusion and mass transfer during epoxidation reactions.

The compliance assessment summarized in Table 4 consolidates these observations. MB meets all evaluated specifications, demonstrating better overall conformity and physicochemical stability. In contrast, EB shows slight noncompliance in moisture content and viscosity, reinforcing the need for stricter control of these parameters when ethyl esters are used as substrates. Nevertheless, both biodiesels are suitable for experimental application and subsequent epoxidation studies, as discussed in this work.

Overall, the data presented in Tables 3 and 4 corroborate the discussion of Section 3.2, indicating that the observed differences between MB and EB arise primarily from intrinsic characteristics of the alcohol moiety rather than deficiencies in the transesterification process itself. These differences are particularly relevant when considering the selectivity and efficiency of the epoxidation reactions discussed in the following sections.

### 3.3 ^1^H NMR Analysis

#### 3.3.1 Characterization of WCO, MB, and EB

The ^1^H NMR spectra obtained for WCO, MB, and EB allowed evaluation of the structural conversion of triacylglycerides and the formation of the corresponding esters. In the WCO spectrum, characteristic signals of triacylglycerides were observed at δ 4.11–4.31 ppm, attributed to the −CH_2_–O–CO– protons of glycerol, as well as multiplets at δ 5.28–5.35 ppm corresponding to unsaturations (−CH=CH−). After transesterification, these signals remained only as residual features in the biodiesel spectra, as shown in Figure 4 (see Supporting Information for details).

**Figure 4:**
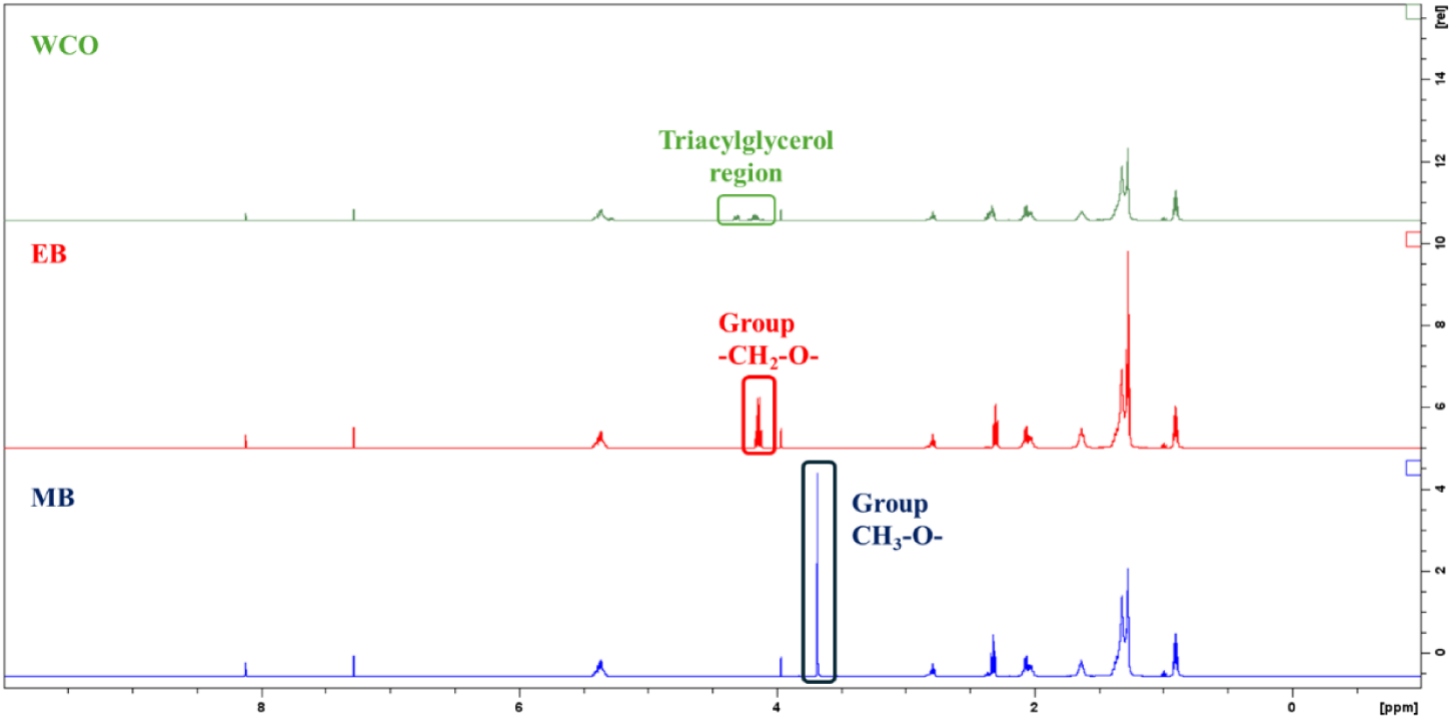
Overlay of ^1^H NMR spectra of WCO, MB, and EB.

The MB spectrum shows an intense singlet at δ 3.66 ppm, characteristic of methoxy protons (−OCH_3_), and signals of terminal methyl groups (ω–CH_3_) at δ 0.80–0.90 ppm. In contrast, the EB spectrum displays a quartet at δ 4.12 ppm, corresponding to −CH_2_–O– protons, and a triplet at δ 1.25 ppm attributed to the terminal methyl protons (−CH_3_) of the ethoxy fragment (−CH_2_–CH_3_). These signals confirm the formation of the respective methyl and ethyl esters.

The conversions of MB and EB were determined as described in Section 2.6.1, using eqs 1 and 2, based on the ratios between the integrals of the characteristic signals of each ester. Integrals were obtained in the regions δ 3.60–3.72/0.75–1.05 ppm for MB and δ 4.06–4.18/0.75–1.05 ppm for EB, as reported in Tables 5 and 6.

**Table 5:** Integral Area and Conversion of MB

sample	area [*I*_OCH_3__]	area [*I*_ω‑CH_3__]	conv. [%]^a^
MB1	70.59	84.30	
MB2	74.15	88.48	83.80 ± 0.17
MB3	73.90	87.90	

a It is the average of the conversions.

**Table 6:** Integral Area and Conversion of EB

SAMPLE	area [*I*_OCH_2__]	area [*I*_ω‑CH_3__]	conv. [%]^a^
EB1	45.55	72.75	
EB2	45.26	72.70	93.91 ± 0.31
EB3	49.80	79.53	

a It is the average of the conversions.

The average conversions calculated were 83.80 ± 0.17% for MB and 93.91 ± 0.31% for EB, indicating higher efficiency of the ethyl route for ester formation, while the methyl route showed lower conversion under the evaluated experimental conditions.

Although the ^1^H NMR spectra of the biodiesels still exhibited residual signals attributed to mono-, di-, or triacylglycerides, the high conversion values obtained indicate predominance of the formed alkyl esters. Thus, the reported percentages represent the relative molar quantification of esters detected by NMR and should not be interpreted as absolute structural conversion of triacylglycerides. This distinction is widely discussed in the literature and arises from experimental and kinetic factors, including (i) small variations in integration windows and baseline correction, which may affect integral accuracy;^36^ (ii) formation of residual mono- and diglycerides, which reduce the fraction of free esters, as described by Gerpen^27^ and associated with the polar nature of the ethyl route; and (iii) reversible equilibrium effects and moisture, which affect the efficiency of ethyl transesterification.^41^ Accordingly, the values obtained by ^1^H NMR express the actual spectroscopic yield of ester formation, consistent with analytical limitations and the expected reaction behavior of methyl and ethyl systems derived from waste oils.

#### 3.3.2 Characterization of Epoxidation Reactions of Transesterified Methyl (MB) and Ethyl (EB) Esters

The epoxidation of methyl and ethyl esters derived from waste cooking oil biodiesel (MB and EB) was evaluated by quantitative ^1^H NMR (q ^1^H NMR), using the terminal methyl group (δ 0.85–0.92 ppm) and oxirane protons (δ 2.87–3.15 ppm) for quantification.

The progressive decrease of olefinic signals (δ 5.28–5.41 ppm), accompanied by the appearance of oxirane resonances, confirmed the conversion of unsaturations into epoxide groups.

Six peracetic acid (PAA) concentrations were evaluated in the production of MB and EB epoxides. Table 7 lists the epoxide sample codes and the corresponding PAA concentrations used in the reactions.

**Table 7:** Epoxide Samples Produced with Their Respective PAA Concentrates

MB epoxides^a^	EB epoxides^b^	PAA conc. (%)^c^
MB451	EB451	1.65
MB11	EB11	1.67
MB32	EB32	2.00
MB315	EB315	2.08
MB31	EB31	2.16
MB15	EB15	15.00

a Epoxides of methyl biodiesel.

b Epoxides of ethyl biodiesel.

c Peracetic acid concentration calculated according to Evonik Operations GmbH.^34^

##### 3.3.2.1 Epoxidation of Methyl Esters (MB)

The ^1^H NMR spectra in Figure 5 clearly show the formation of oxirane signals (δ 2.87–3.15 ppm, 2H) and partial or total reduction of olefinic resonances (δ 5.28–5.41 ppm) (see Supporting Information for details).

**Figure 5:**
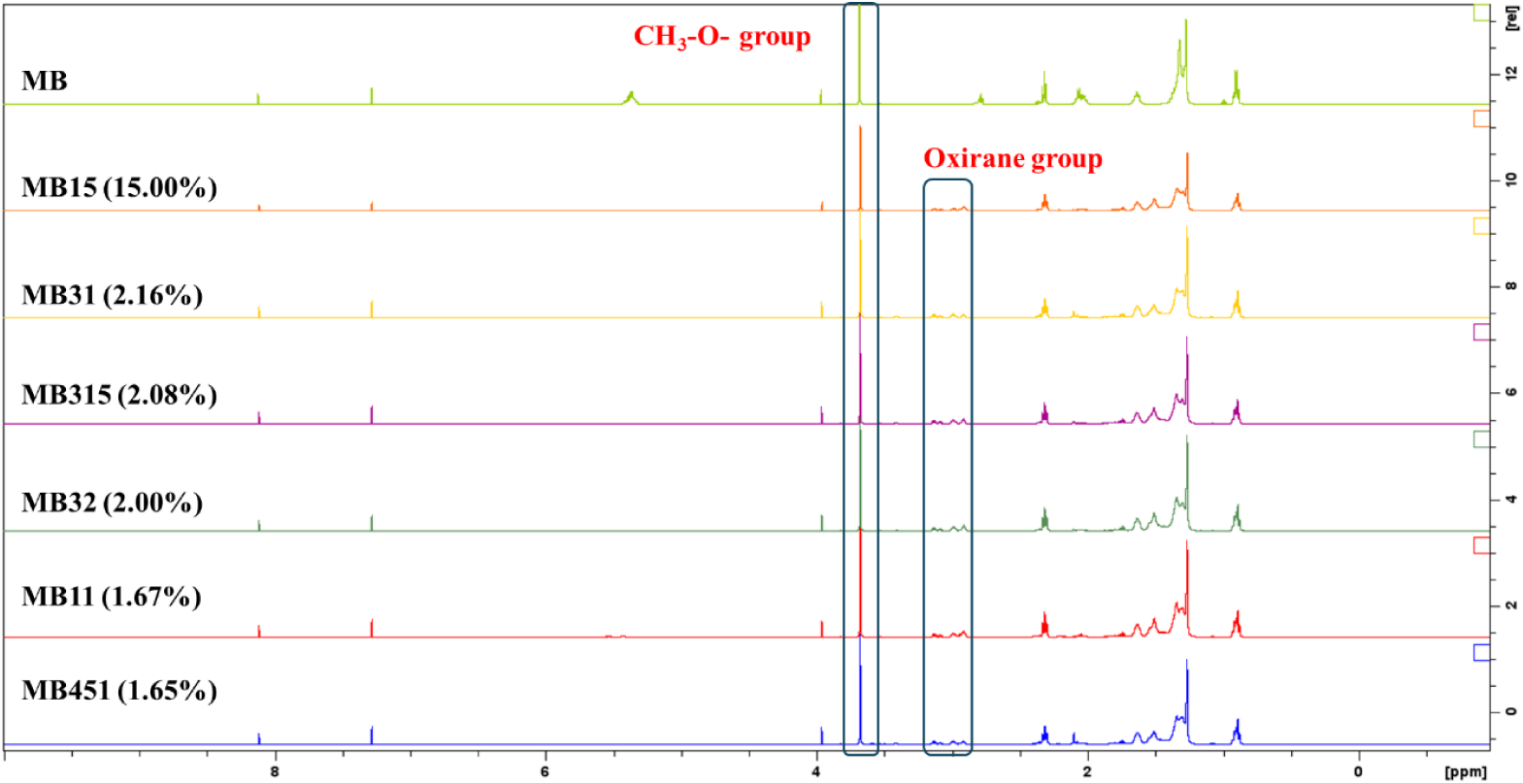
^1^H NMR (500 MHz) spectra of epoxidized methyl ester samples (MB) obtained using different concentrations of peracetic acid: MB451 (1.65%), MB11 (1.67%), MB32 (2.00%), MB315 (2.08%), MB31 (2.16%), and MB15 (15.00%).

The ratio between the integrals of the terminal methyl group (ω–CH_3_) and the oxirane protons, normalized by the average initial number of double bonds (DB_0_ = 1.2843), allowed the calculation of epoxidation conversion according to eqs 3 and 4. The NMR integration areas used in these calculations are provided in the Supporting Information, while the calculated conversion values are summarized in Table 8.

**Table 8:** Epoxidation Conversion of Methyl Biodiesel (MB) Determined by q ^1^H NMR^c^

sample	PAA conc. (%)^a^	conv. [%]^b^
MB451	1.65	64.16 ± 0.20^c^
MB11	1.67	74.25 ± 0.04^a^
MB32	2.00	71.83 ± 0.14^b^
MB315	2.08	71.65 ± 0.19^b^
MB31	2.16	59.83 ± 0.11^d^
MB15	15.00	63.97 ± 0.18^c^

a Peracetic acid concentration calculated according to Evonik Operations GmbH.^34^

b It is the average of the conversions calculated according to the methodology of Aerts and Jacobs (2004), adapted by Xia et al. (2016).

c Different letters indicate statistically significant differences among mean conversion values, according to the Tukey test (*p* < 0.05).

Statistical significance of the observed differences in epoxidation conversion of methyl esters was evaluated by one-way analysis of variance (ANOVA). The ANOVA results revealed an extremely high *F* value (*F* = 3936.82) associated with a very low *p* value (*p* = 7.49 × 10^–19^), which is several orders of magnitude below the adopted significance level (α = 0.05). These results clearly demonstrate that epoxidation conversion of methyl esters is strongly influenced by experimental conditions, particularly peracetic acid concentration.

The magnitude of the F statistic indicates that between-group variance is much greater than within-group variance, confirming that the observed differences in epoxidation conversion are not due to random experimental fluctuations but rather to systematic changes in reaction conditions.

The Tukey post hoc test (*p* < 0.05) allowed identification of statistically distinct groups among the evaluated conditions. The highest conversion was obtained for MB11, which differed significantly from all other experimental conditions. Conversions observed for MB32 and MB315 were statistically equivalent to each other and significantly higher than those obtained for MB451 and MB15. The lowest conversion was observed for MB31, which differed significantly from all other groups. Although peracetic acid concentration is the main factor governing epoxidation conversion, the slightly superior performance observed for MB15 relative to MB31 may be associated with secondary experimental variations, such as residual fluctuations in biodiesel moisture content, stirring efficiency, or effective oxidant consumption by parallel reactions, which affect oxirane ring stability without altering the overall observed trend.^18^

The strong statistical discrimination among experimental conditions, evidenced by the high F value and extremely low *p* value, supports the mechanistic interpretation of an intermediate operational range associated with higher average epoxidation conversions. Intermediate oxidant concentrations favor epoxide formation, whereas lower or excessive concentrations lead to reduced conversion, likely due to incomplete epoxidation or increased occurrence of secondary reactions such as oxirane ring opening.

These statistical trends are consistent with the integral profiles obtained by quantitative ^1^H NMR. Samples processed at intermediate peracetic acid concentrations exhibited a pronounced increase in oxirane proton integrals accompanied by a significant reduction in olefinic signals, indicating efficient conversion of C=C bonds into epoxide groups.

In contrast, lower oxidant concentrations (1.65%) resulted in lower epoxidation efficiency due to incomplete oxidation of internal double bonds, while higher oxidant concentrations (≥2.16%) led to decreased conversion, attributed to competitive secondary reactions such as oxirane ring opening and secondary oxidation processes. This behavior is statistically corroborated by the one-way ANOVA results, confirming that the observed differences among experimental conditions are systematic rather than random.

The Tukey post hoc test further demonstrated that the optimal epoxidation range for methyl esters lies between 1.67% and 2.08% peracetic acid, where the highest conversions were obtained and no statistically significant differences were observed between MB32 and MB315. Outside this range, statistically significant losses in conversion were detected.

Similar trends have been reported in epoxidation systems of fatty substrates, in which increasing oxidant concentration enhances epoxide formation up to a limit, beyond which parallel reactions such as oxirane ring opening and secondary oxidation reduce selectivity and apparent conversion.^13^ Related behavior has been discussed by Hájek et al.^12^ and Cogliano et al.,^17,44^ who demonstrated that increased acidity or oxidant severity enhances the susceptibility of the oxirane ring to acid-catalyzed opening reactions, leading to reduced epoxide selectivity and apparent conversion.

Although statistically significant differences in conversion values were observed among the investigated conditions, the epoxidation of methyl esters did not exhibit a strong monotonic dependence on peracetic acid (PAA) concentration within the range of 1.65–15.00%. Instead, comparable conversion levels were obtained across a relatively wide interval of oxidant concentrations, suggesting that, under the reaction conditions employed, PAA was not the limiting factor governing epoxide formation. This behavior indicates the presence of a broad operational window in which variations in oxidant concentration have a limited impact on the overall conversion, rather than a sharply defined optimal PAA concentration. Such reduced sensitivity to oxidant concentration may be associated with the excess of PAA already at the lowest level investigated and/or with the contribution of mass transfer phenomena and parallel reactions, which can attenuate the effect of increasing oxidant availability.

Therefore, within the investigated concentration range, variation in peracetic acid concentration does not constitute an effective control parameter for enhancing methyl ester epoxidation conversion, as the process exhibits limited sensitivity to oxidant levels under the applied conditions. Ethyl ester epoxidation (EB) is discussed separately in Section 3.3.2.2 due to its distinct behavior and higher experimental variability.

##### 3.3.2.2 Epoxidation of Ethyl Esters (EB)

The EB samples (EB451 to EB15) exhibited spectral evolution similar to that observed for MB, as shown in Figure 6 (see Supporting Information for details).

**Figure 6:**
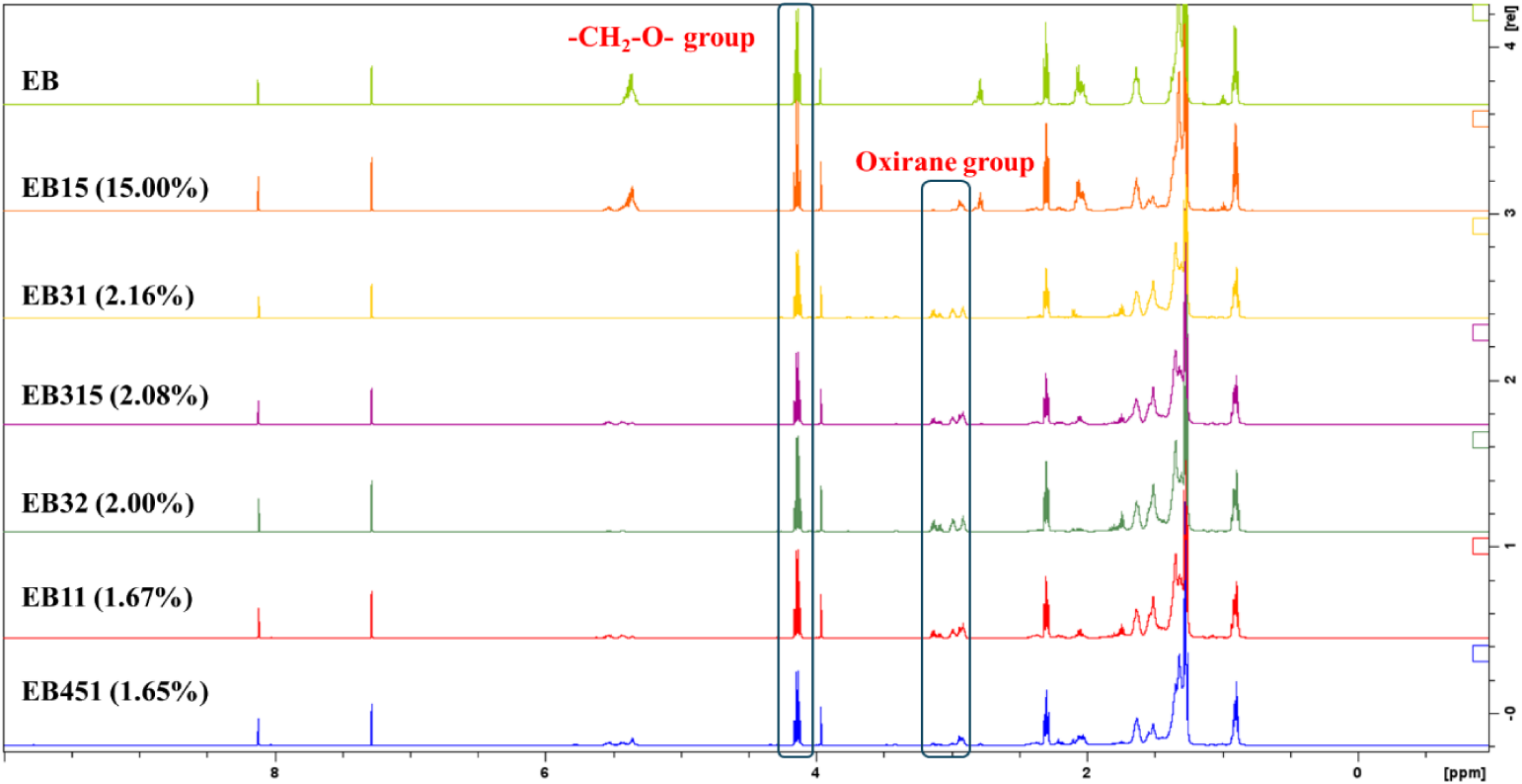
^1^H NMR (500 MHz) spectra of epoxidized ethyl ester samples (EB) obtained using different concentrations of peracetic acid: EB451 (1.65%), EB11 (1.67%), EB32 (2.00%), EB315 (2.08%), EB31 (2.16%), and EB15 (15.00%).

Oxirane signals appeared at δ 2.87–3.15 ppm, while the decrease of olefinic resonances (δ 5.28–5.56 ppm) confirmed epoxide formation. The ethoxy group signal (δ 4.12 ppm) remained unchanged, serving as a structural marker of ester integrity.

The ratio between the integrals of the terminal methyl group and oxirane protons, normalized by the average number of double bonds (DB_0_ = 1.2843), allowed calculation of epoxidation conversion according to eqs 3 and 4. The NMR integration areas used in these calculations are provided in the Supporting Information, while the calculated conversion values are summarized in Table 9.

**Table 9:** Epoxidation Conversion of Ethyl Biodiesel (EB) Determined by q ^1^H NMR^c^

sample	PAA conc. (%)^a^	conv. [%]^b^
EB451	1.65	40.10 ± 1.16^e^
EB11	1.67	76.46 ± 0.13^b^
EB32	2.00	80.62 ± 0.11^a^
EB315	2.08	73.32 ± 0.12^c^
EB31	2.16	69.42 ± 0.13^d^
EB15	15.00	27.08 ± 0.08^f^

a Peracetic acid concentration calculated according to Evonik Operations GmbH.^34^

b It is the average of the conversions calculated according to the methodology of Aerts and Jacobs (2004), adapted by Xia et al. (2016).

c Different letters indicate statistically significant differences among mean conversion values, according to the Tukey test (*p* < 0.05).

Statistical significance of the observed differences in epoxidation conversion of ethyl esters was evaluated by one-way ANOVA. The results revealed an extremely high *F* value (*F* = 6261.02) associated with a very low *p* value (*p* = 4.64 × 10^–20^), well below the significance level (α = 0.05), unequivocally demonstrating that epoxidation conversion is strongly dependent on experimental conditions, particularly peracetic acid concentration.

The high F value indicates that between-group variance is much greater than within-group variance, confirming that the observed differences are not attributable to experimental noise or random fluctuations, but rather to systematic changes in reaction conditions. The Tukey post hoc test (*p* < 0.05) confirmed that all experimental conditions differ statistically from one another, indicating a clear hierarchy of epoxidation efficiency among the evaluated ethyl ester systems.

The highest conversion was obtained for EB32, followed by EB11 and EB315, whereas intermediate conversion was observed for EB31. Significantly lower conversions were obtained for EB451 and EB15, highlighting the detrimental effect of insufficient or excessive oxidant concentration on epoxidation efficiency.

The robustness of the ANOVA results, reflected by the exceptionally low p value and high F statistic, reinforces the mechanistic interpretation proposed in this study. The existence of statistically distinct groups under all conditions suggests a narrow optimal range for epoxidation efficiency in ethyl esters, in which oxidant concentration plays a decisive role in balancing epoxide formation and secondary reactions such as oxirane ring opening.

At low oxidant concentration (1.65% PAA; EB451), reduced conversion (∼40.10%) was observed, consistent with oxidant limitation. Conversion increased within the optimal range (1.67–2.08% PAA), reaching a maximum at 2.00% (EB32; ∼80.62%), followed by EB11 (∼76.46%) and EB315 (∼73.32%). Above this range, conversion decreased again (2.16% PAA; BE31 ∼69.42%) and dropped sharply at 15% PAA (EB15; ∼27.08%), consistent with increased contribution of parallel reactions such as oxirane ring opening.

The differences observed between methyl and ethyl ester epoxidation conversions cannot be attributed to intrinsic electronic effects at the C=C bond, since the electronic distinction between the −OCH_3_ and −OCH_2_CH_3_ groups is negligible at the alkene moiety several bonds away. Instead, the distinct epoxidation behavior of ethyl esters is more consistently interpreted in terms of indirect effects related to the reaction medium. In particular, differences in global polarity, phase behavior, and miscibility between the ester substrate and the polar oxidizing phase may influence oxidant accessibility and mass transfer at the reaction interface.^45^ Such effects can lead to apparent differences in conversion, especially at early reaction stages, without implying enhanced intrinsic reactivity of the double bond. Therefore, the observed variations between methyl and ethyl ester epoxidation are more reasonably associated with differences in microenvironment and transport phenomena rather than direct electronic activation by the ethoxy group.

These interpretations are further corroborated by the Tukey test results, which confirmed statistically distinct conversion levels for all evaluated ethyl ester systems.

Accordingly, Figure 7 provides a comparative visualization of the evolution of the mean integrated areas of oxirane (I_epoxide_ and olefinic I_C=C_) signals obtained by quantitative ^1^H NMR for epoxidized methyl (MB) and ethyl (EB) biodiesel samples. Within the effective reaction window, increasing peracetic acid concentration is associated with an increase in the relative area of the oxirane signal, accompanied by a concomitant decrease in the olefinic signal area, reflecting the progressive conversion of C=C double bonds into oxirane rings. This inverse relationship is consistent with the conversion values reported in Tables 8 and 9 and supports the reliability of the NMR-based quantification approach. At intermediate oxidant concentrations, this behavior is more pronounced, indicating higher apparent epoxidation efficiency, whereas deviations observed at low and excessive peracetic acid levels may be associated, respectively, with incomplete oxidation of unsaturations or an increased contribution of parallel reactions, in agreement with trends commonly reported in the literature.

**Figure 7:**
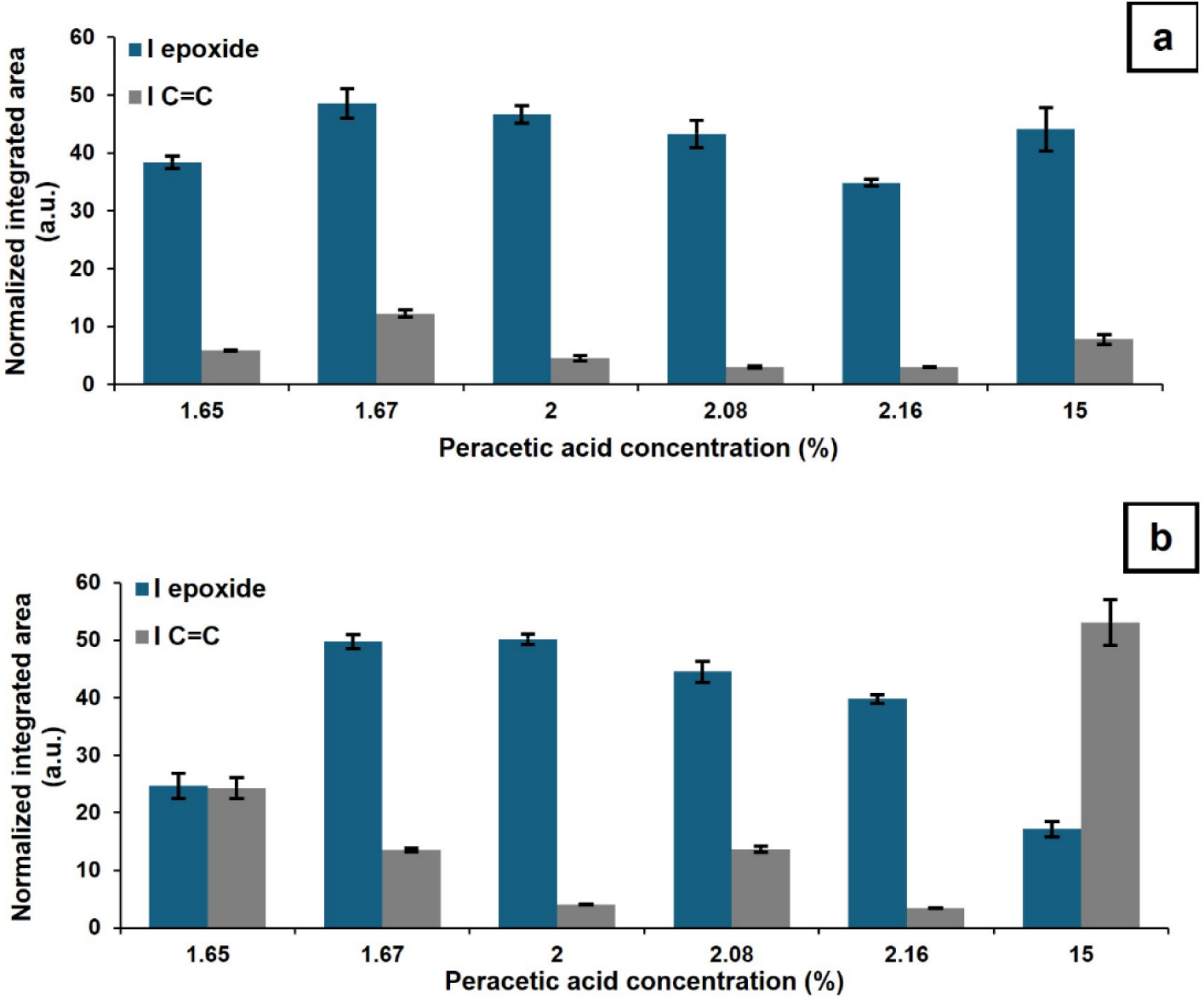
Variation of the mean integrated areas of oxirane (*I*_epoxide_) and olefinic (*I*_C=C_) signals obtained by quantitative ^1^H NMR for epoxidized biodiesel samples as a function of peracetic acid concentration: (a) methyl biodiesel (MB) and (b) ethyl biodiesel (EB). Error bars represent the standard deviation of triplicate experiments.

#### 3.3.3 Comparative Analysis and Mechanistic Discussion

The epoxidation of methyl (MB) and ethyl (EB) biodiesel exhibited a bell-shaped (parabolic) dependence of conversion on peracetic acid concentration, as illustrated in Figure 8. This behavior reflects the balance between epoxide formation and degradation pathways.

**Figure 8:**
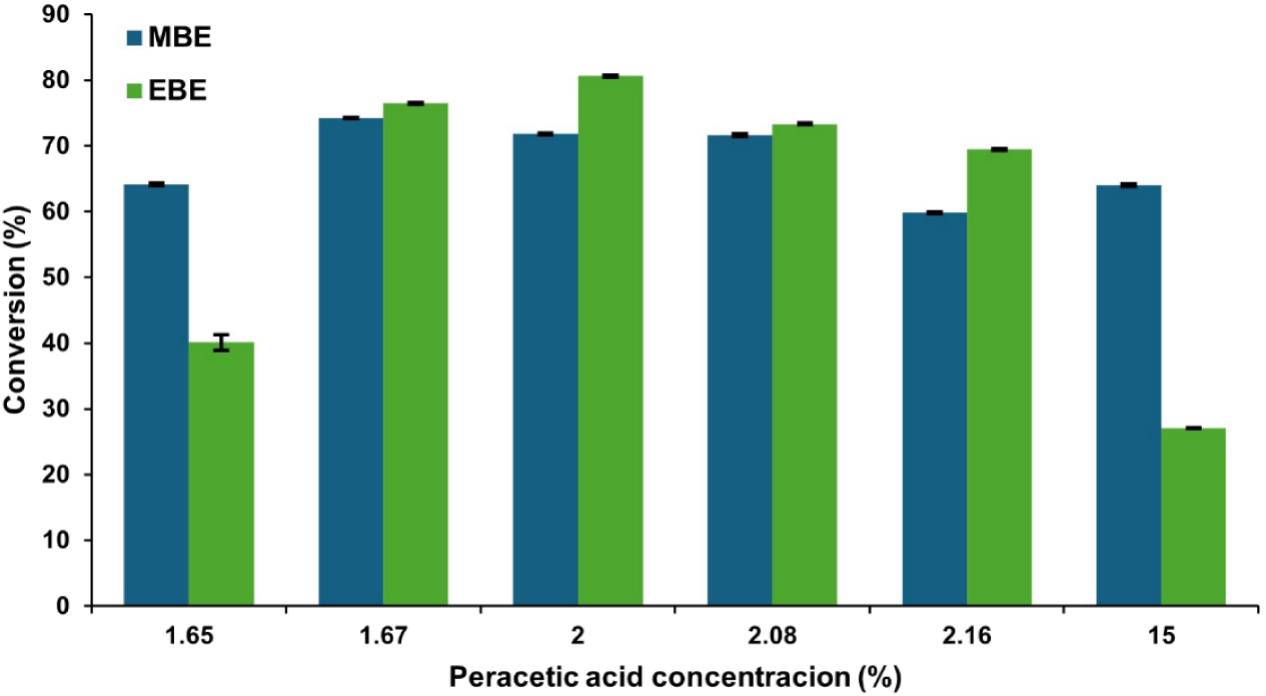
Conversion of methyl (MBE) and ethyl (EBE) biodiesel epoxides as a function of peracetic acid concentration.

Considering the statistically significant differences established in the previous sections for methyl esters, the highest conversion was obtained at 1.67% peracetic acid (74.25%), whereas ethyl esters reached their maximum conversion at 2.00% (80.62%). These results indicate that although both systems show similar parabolic trends, the optimal oxidant concentration differs slightly depending on the ester group, reflecting a balance between peracid reactivity and oxirane ring stability.

At lower oxidant concentrations, limited availability of reactive oxygen species restricts epoxidation extent, whereas at higher concentrations excess acidity promotes oxirane ring protonation and subsequent nucleophilic opening, leading to diol formation and secondary oxidation products.^15,18,43^ This effect explains the reduced conversion observed for both systems at 2.16% and, more markedly, at 15.00% peracetic acid.

These comparative trends are corroborated by the statistical analyses presented in Sections 3.3.2.1 and 3.3.2.2, which confirmed significant differences between optimal and nonoptimal conditions for both ester systems.

Epoxidation proceeds via electrophilic oxygen transfer from peracetic acid to the C=C bond through a concerted Prilezhaev-type mechanism, producing the oxirane ring and acetic acid as a byproduct. Residual olefinic signals around δ 5.3 ppm observed in MB and EB epoxides indicate incomplete conversion of internal double bonds, likely due to steric hindrance and diffusional limitations.^38^ Comparatively, the maximum conversions of ∼74.25% (MB) and ∼80.62% (EB) indicate efficient epoxidation under mild conditions (45 ± 5 °C), consistent with green chemistry principles.

Ethyl esters exhibited higher maximum conversion than methyl esters under comparable conditions; however, this behavior cannot be attributed to intrinsic electronic effects of the ester alkoxy group on the C=C bond. Instead, the observed differences are more consistently explained by indirect physicochemical factors, such as differences in phase behavior, miscibility with the polar oxidizing medium, and mass transfer at the reaction interface.^45^ These parameters influence oxidant accessibility and apparent reaction rates without implying enhanced intrinsic reactivity of the unsaturated moieties. This interpretation is consistent with the absence of a direct electronic interaction between the ester group and the alkene several bonds away.

The conversion levels obtained are consistent with values of 65–80% reported by Xia et al.^18^ and Cogliano et al.^17^ for biodiesels with similar degrees of unsaturation, reinforcing the reproducibility of the proposed methodology. Additionally, Kurańska et al.^46^ reported conversion close to 81% of double bonds when epoxidizing waste cooking oil with *in situ* peracid, although with limited selectivity (∼47%) due to side reactions.

## 4 Conclusion

This study demonstrated the applicability of quantitative ^1^H NMR (q ^1^H NMR) as a robust and reliable tool for monitoring the epoxidation of biodiesel-derived methyl (MB) and ethyl (EB) esters obtained from waste cooking oil. The proposed methodology enabled accurate determination of epoxidation conversion, providing consistent results across a wide range of experimental conditions and allowing meaningful comparison between ester types.

For methyl esters, although statistically significant differences in conversion values were identified, the epoxidation process did not exhibit a strong monotonic dependence on peracetic acid (PAA) concentration within the investigated range. Comparable conversion levels were obtained across a broad interval of oxidant concentrations, indicating a wide operational window rather than a sharply defined optimal PAA concentration. These results suggest that, under the conditions employed, variations in oxidant concentration are not the primary factor governing epoxidation efficiency.

The comparative analysis between methyl and ethyl ester epoxidation confirms that the observed differences in conversion behavior arise from system-level effects associated with reaction medium properties, such as phase behavior, oxidant accessibility, and mass transfer phenomena. Importantly, no intrinsic electronic activation of the C=C bond by the ester alkoxy group is implied. This interpretation ensures consistency between experimental evidence, statistical analysis, and mechanistic understanding, reinforcing that epoxidation performance is governed by physicochemical and transport-related factors rather than molecular electronic effects.

Overall, the results highlight that epoxidation efficiency in biodiesel-derived esters is governed by a combination of operational conditions and transport-related effects rather than direct electronic effects associated with the alcohol moiety. The findings contribute to a more accurate interpretation of ester epoxidation behavior and support the use of q ^1^H NMR as a valuable analytical approach for process evaluation and optimization in biodiesel upgrading and biolubricant precursor production.

## Supplementary Material



## Notes

Illustrated ^1^H NMR spectra of WCO, MB, EB, and epoxides, as well as the reaction schemes prepared for the reactions in photographic images (PDF)
