# Supplementary material for: Quantitative Analysis of the Epoxidation of Waste Cooking Oil Biodiesel by 1H NMR
Source: ACS Omega. 2026 Jul 6;11(28):41206–17. doi: 10.1021/acsomega.5c11222 (PMC13393197; doi:10.1021/acsomega.5c11222)
Supplement: Supplementary file 1 [file ao5c11222_si_001.pdf]

QUANTITATIVE ANALYSIS OF THE EPOXIDATION OF WASTE COOKING OIL BIODIESEL BY ^{1}H NMR

João F. A. Costa[a,b,#], Leiliane do S. S. de Souza[b], Kidney O. G. Neves[c], Marcos B. Machado[c], Luiz K. C. de Souza[d], Sergio D. Junior[e], Eduardo A. C. Batista[f], Ramon S. B. Ferreira[g], Anderson M. Pereira[b,*], João N. N. Quaresma[a]

[a]Graduate Program in Engineering of Natural Resources of the Amazon (PRODERNA), Institute of Technology, Federal University of Pará (UFPA), Belém, PA 66075-110, Brazil

[b]Department of Agricultural and Soil Engineering, Faculty of Agricultural Sciences, Federal University of Amazonas (UFAM), Manaus, AM 69080-900, Brazil

[c]Nuclear Magnetic Resonance Laboratory (NMRLAB), Analytical Center, Multidisciplinary Support Center, Federal University of Amazonas (UFAM), Manaus, AM 69080-900, Brazil

[d]Department of Chemistry, Institute of Exact Sciences, Federal University of Amazonas (UFAM), Manaus, AM 69080-900, Brazil

[e]Chemical Analysis Center (CAQQAT), School of Technology, Amazonas State University (UEA), Manaus, AM 69050-020, Brazil

[f]Extraction, Applied Thermodynamics, and Equilibrium Laboratory (EXTRAE), School of Food Engineering, University of Campinas (UNICAMP), Campinas, SP 13083-862, Brazil

[g]Center for Social Sciences, Health, and Technology, Federal University of Maranhão (UFMA), Imperatriz, MA 65915-240, Brazil

**Corresponding Author:*

*E-mail: andersonpereira@ufam.edu.br

#CURRENT ADDRESS

#João F. A. Costa: Department of Agricultural and Soil Engineering, Faculty of Agricultural Sciences, Federal University of Amazonas (UFAM), Av. Gen. Rodrigo Octávio No. 6200, South Sector, Coroado I, Manaus, AM 69080-900, Brazil.

Supporting Information

Quantitative Analysis of Waste Cooking Oil Biodiesel Epoxidation by ^{1}H NMR

S1. Apparatus used in the production of biodiesel, peracetic acid, and epoxides

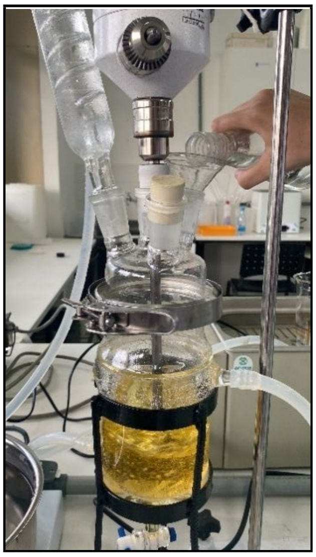

Figure S1. Reactor used in the transesterification experiments.

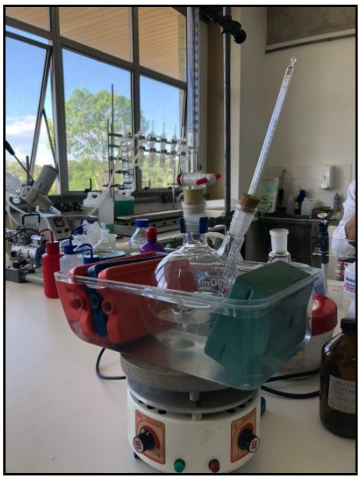

Figure S2. Ice bath with an attached burette used for the production of peracetic acid.

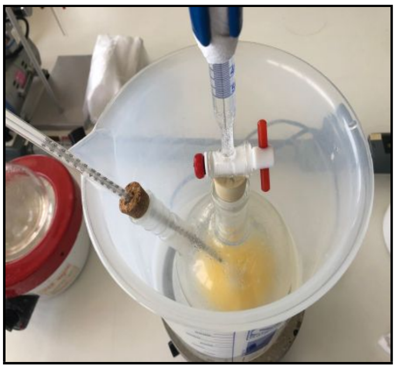

Figure S3. Apparatus used for the epoxidation reactions.

S2. ^{1}H NMR spectra of WCO, MB, and EB

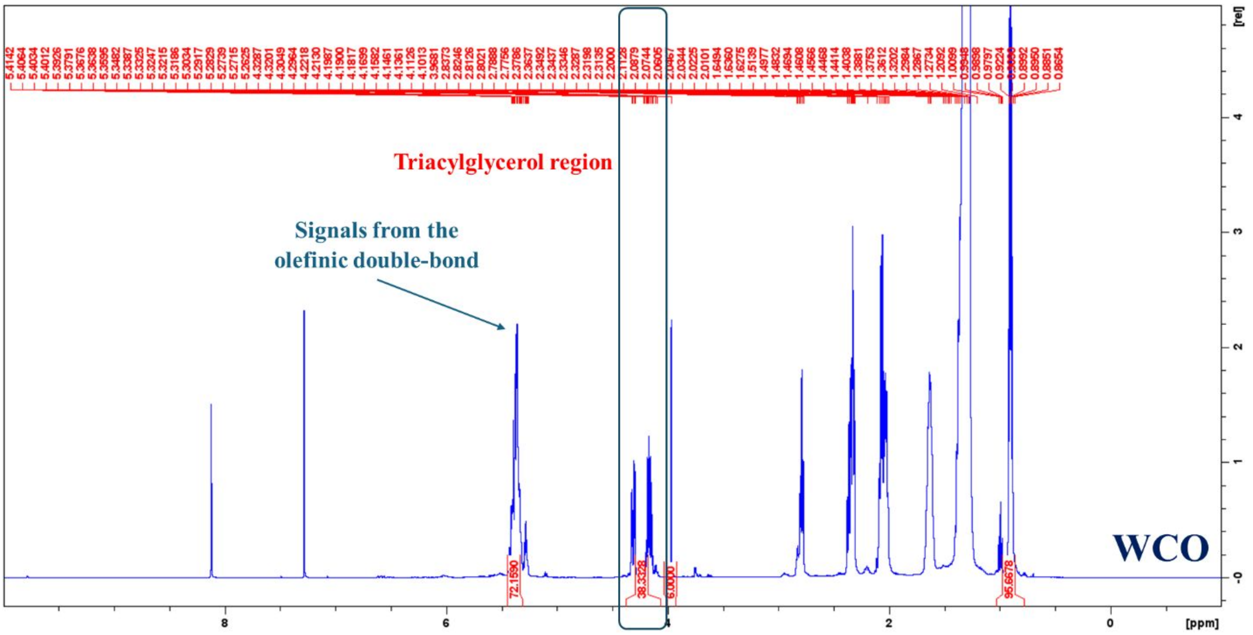

Figure S4. ^{1}H NMR (500 MHz) spectrum of the **WCO** sample in $CDCl_3$.

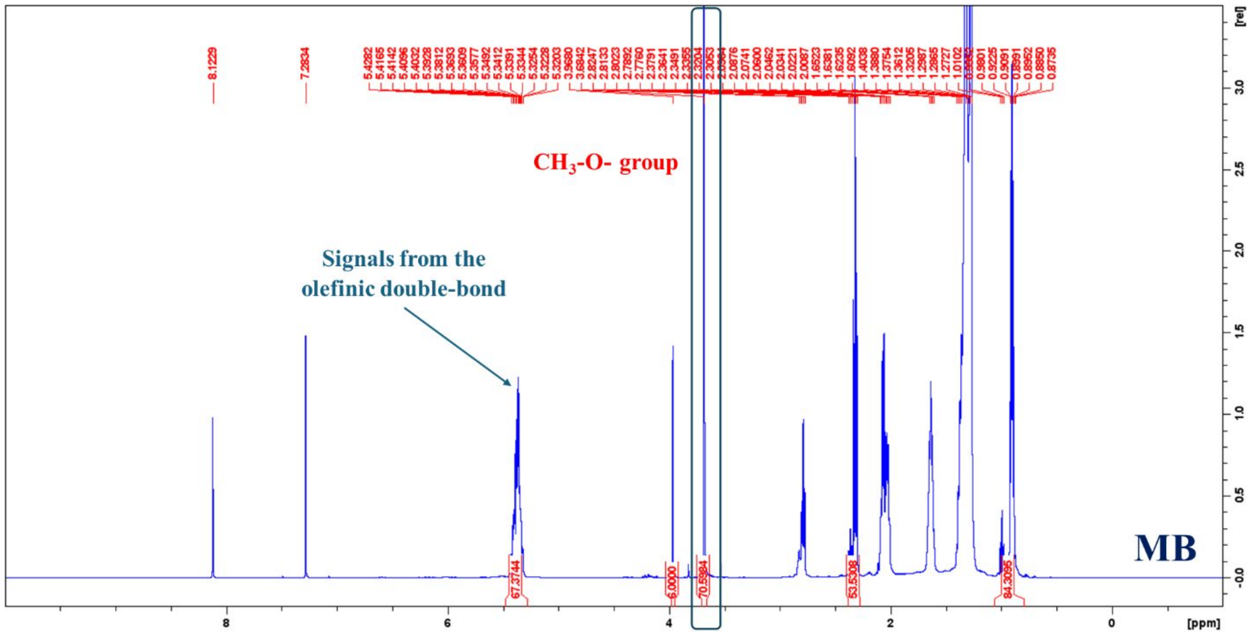

Figure S5. ^{1}H NMR (500 MHz) spectrum of the **MB** sample in $CDCl_3$.

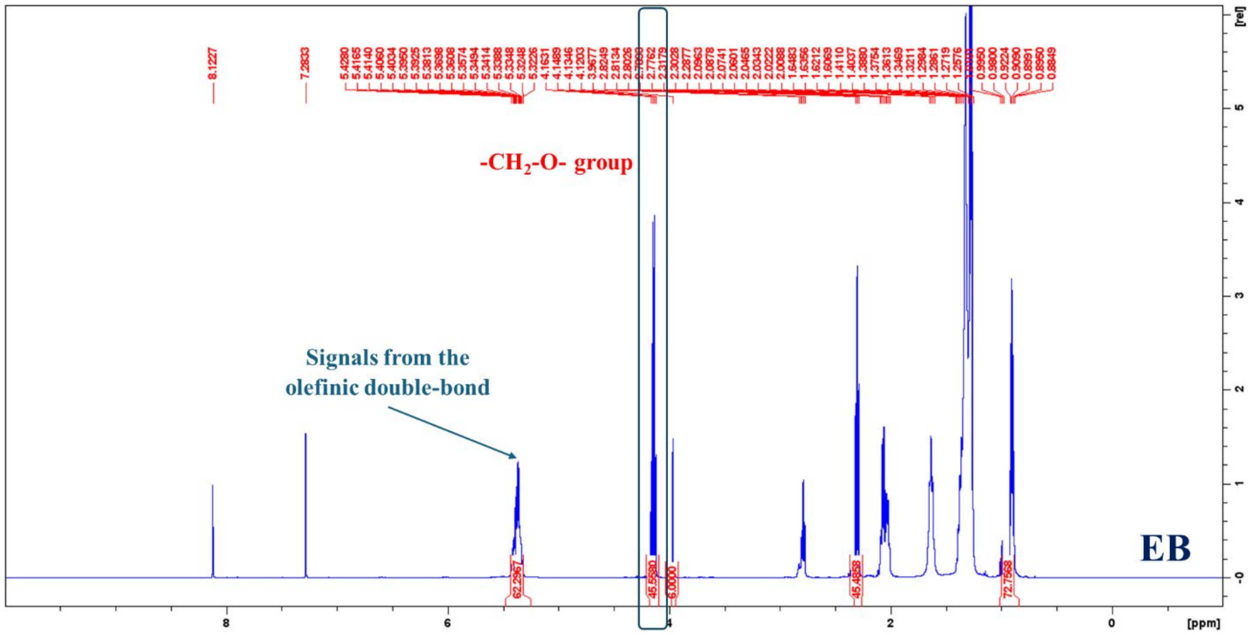

Figure S6. ^{1}H NMR (500 MHz) spectrum of the **EB** sample in $CDCl_3$.

S3. ¹H NMR spectra of the epoxidation reactions of methyl esters derived from MB

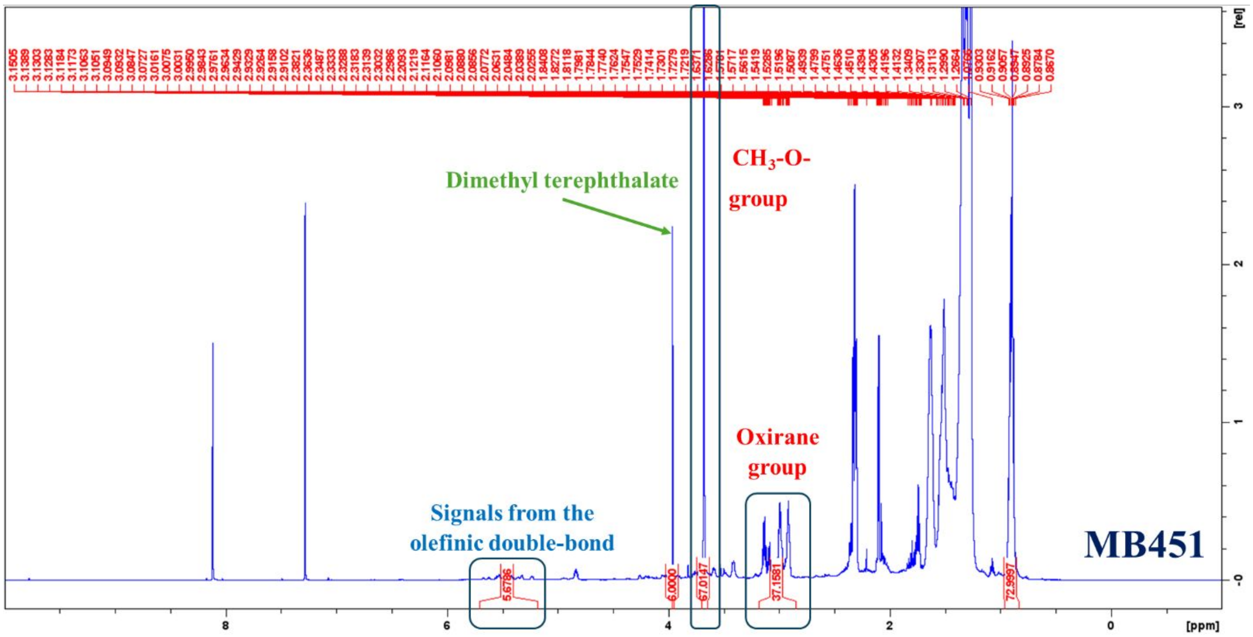

Figure S7. ¹H NMR (500 MHz) spectrum of the M**B451** sample (1.65% PA) in $CDCl_3$.

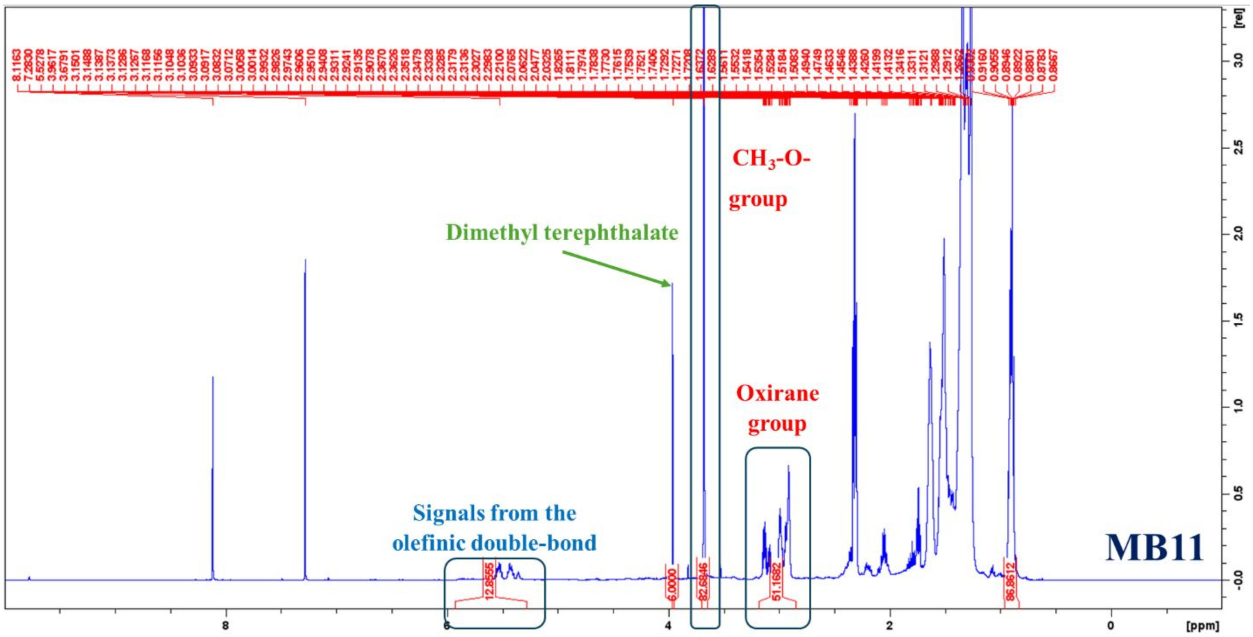

Figure S8. ¹H NMR (500 MHz) spectrum of the M**B11** sample (1.67% PA) in $CDCl_3$.

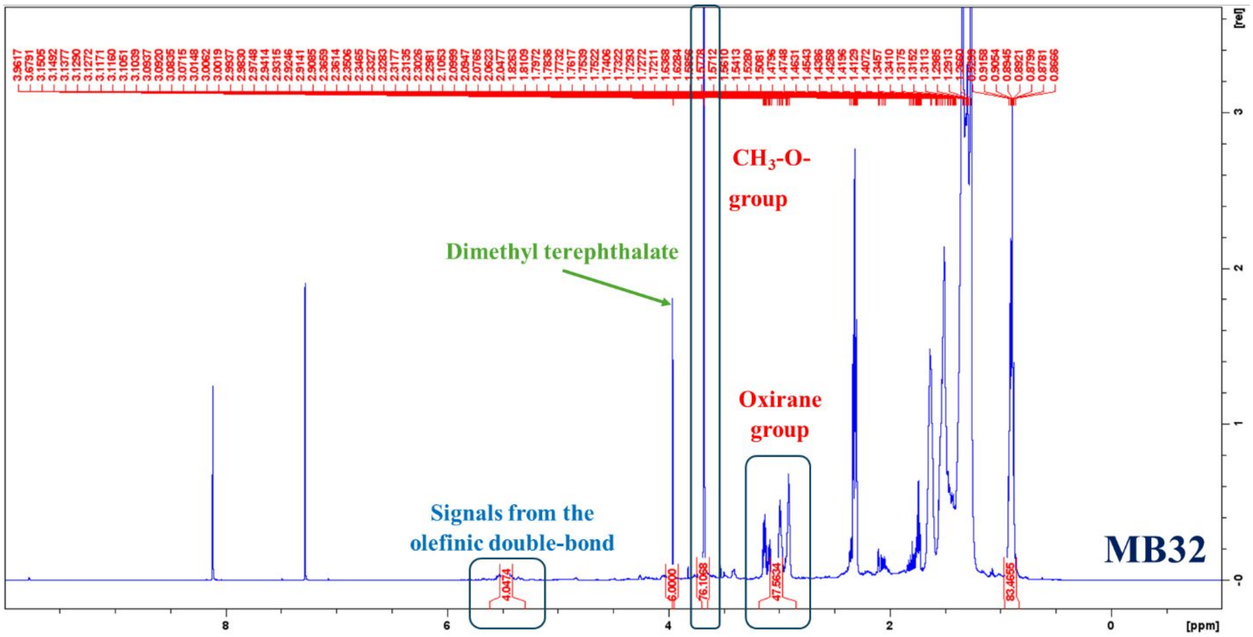

Figure S9. ^{1}H NMR (500 MHz) spectrum of the M**B32** sample (2.00% PA) in $CDCl_3$.

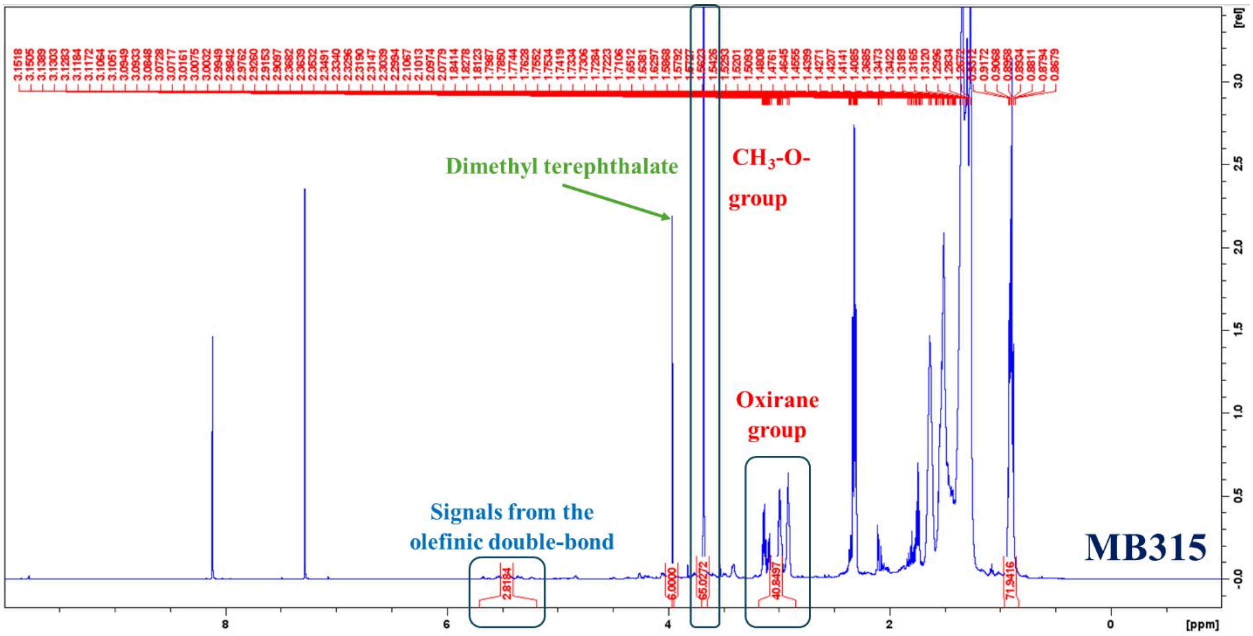

Figure S10. ^{1}H NMR (500 MHz) spectrum of the M**B315** sample (2.08% PA) in $CDCl_3$.

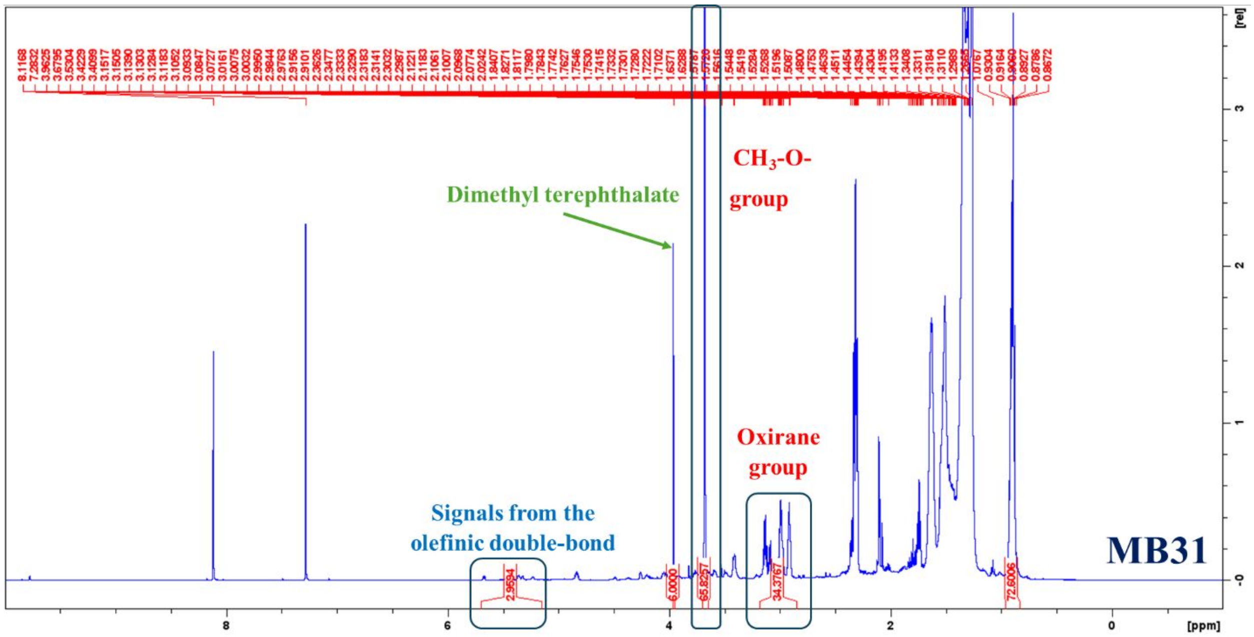

Figure S11. ^{1}H NMR (500 MHz) spectrum of the M**B31** sample (2.16% PA) in $CDCl_3$.

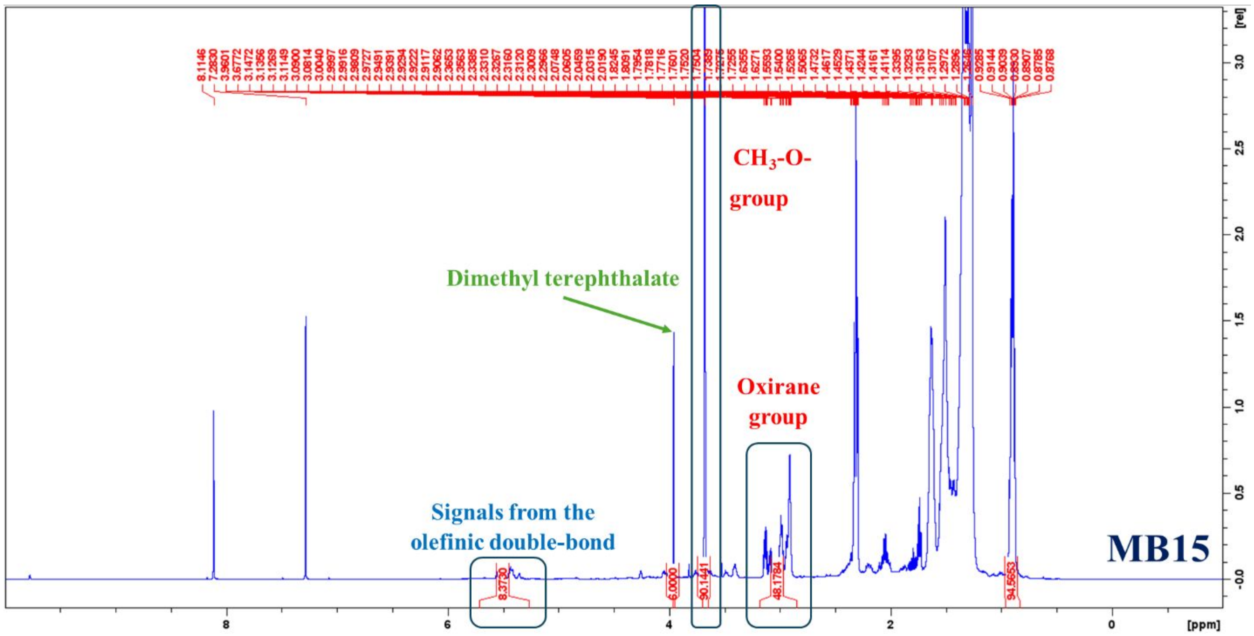

Figure S12. ^{1}H NMR (500 MHz) spectrum of the M**B15** sample (15.00% PA) in $CDCl_3$.

S4. ^{1}H NMR spectra of the epoxidation reactions of ethyl esters derived from EB

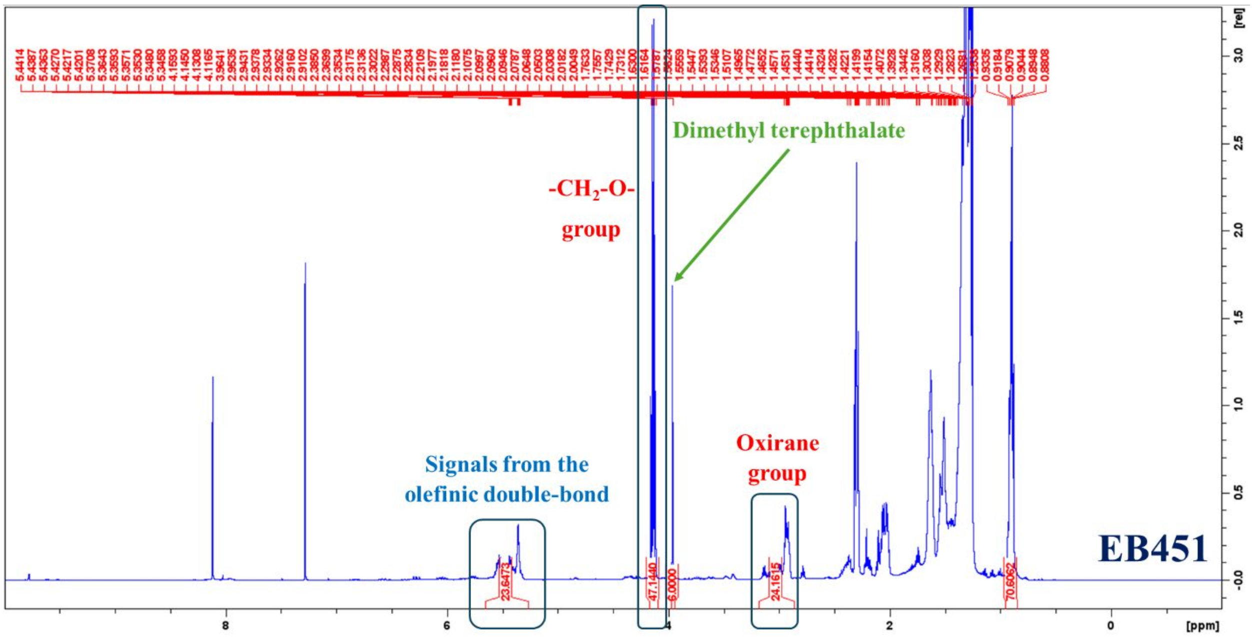

Figure S13. ^{1}H NMR (500 MHz) spectrum of the **EB451** sample (1.65% PA) in $CDCl_3$.

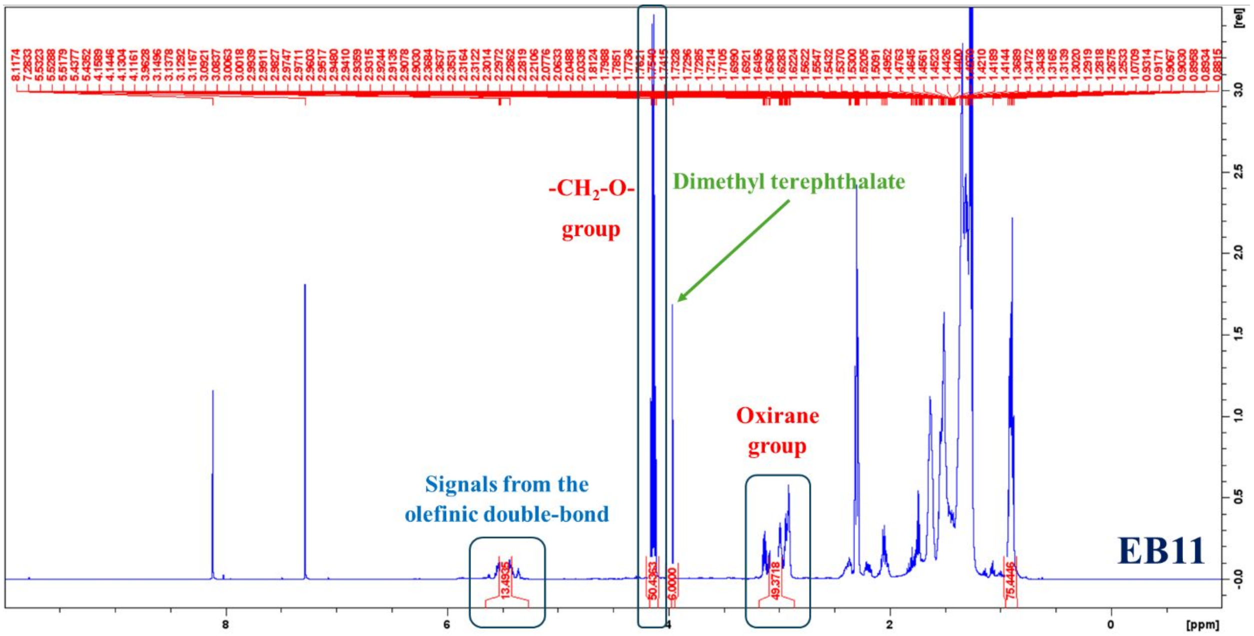

Figure S14. ^{1}H NMR (500 MHz) spectrum of the **EB11** sample (1.67% PA) in $CDCl_3$.

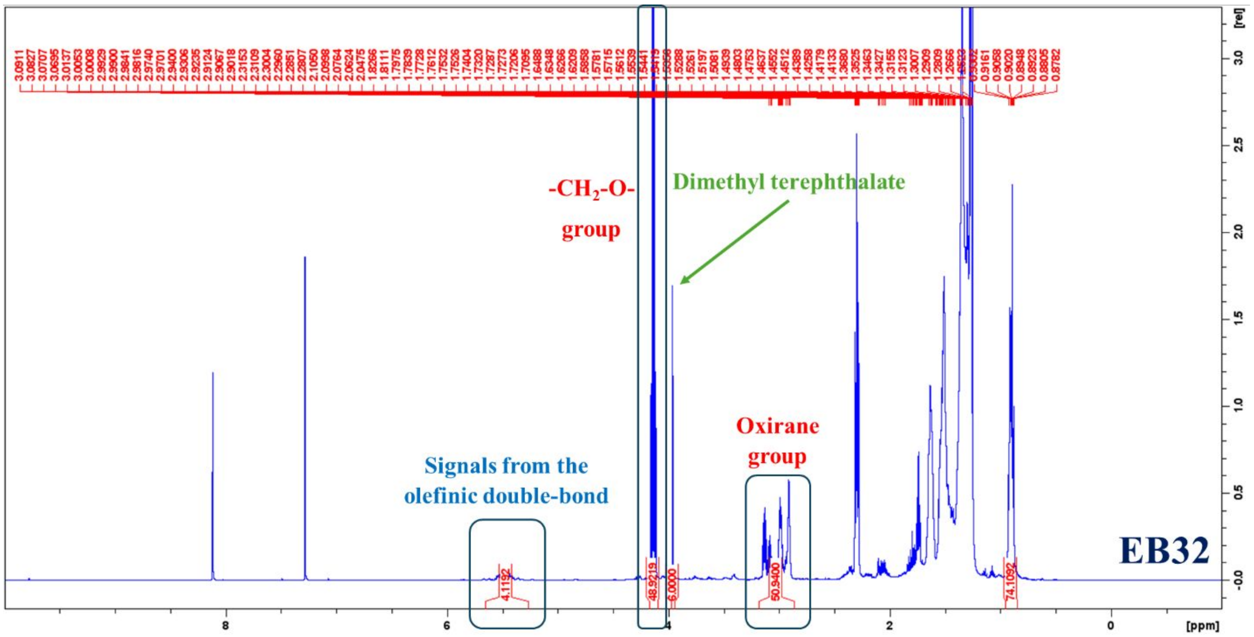

Figure S15. ^{1}H NMR (500 MHz) spectrum of the **EB32** sample (2.00% PA) in $CDCl_3$.

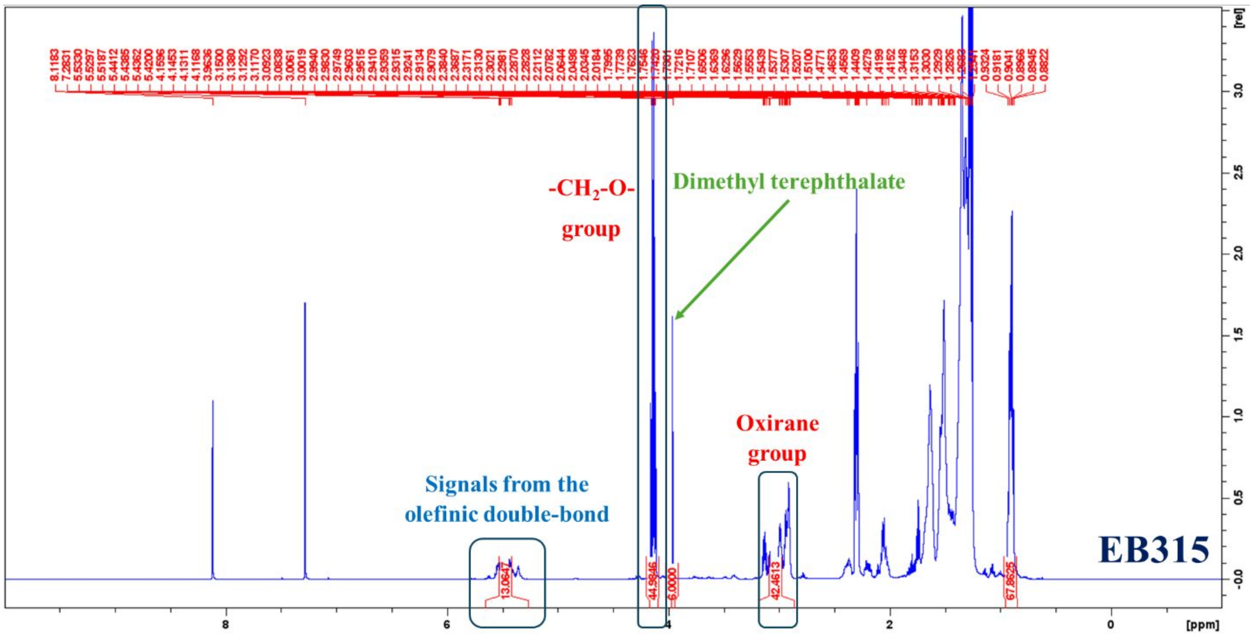

Figure S16. ^{1}H NMR (500 MHz) spectrum of the **EB315** sample (2.08% PA) in $CDCl_3$.

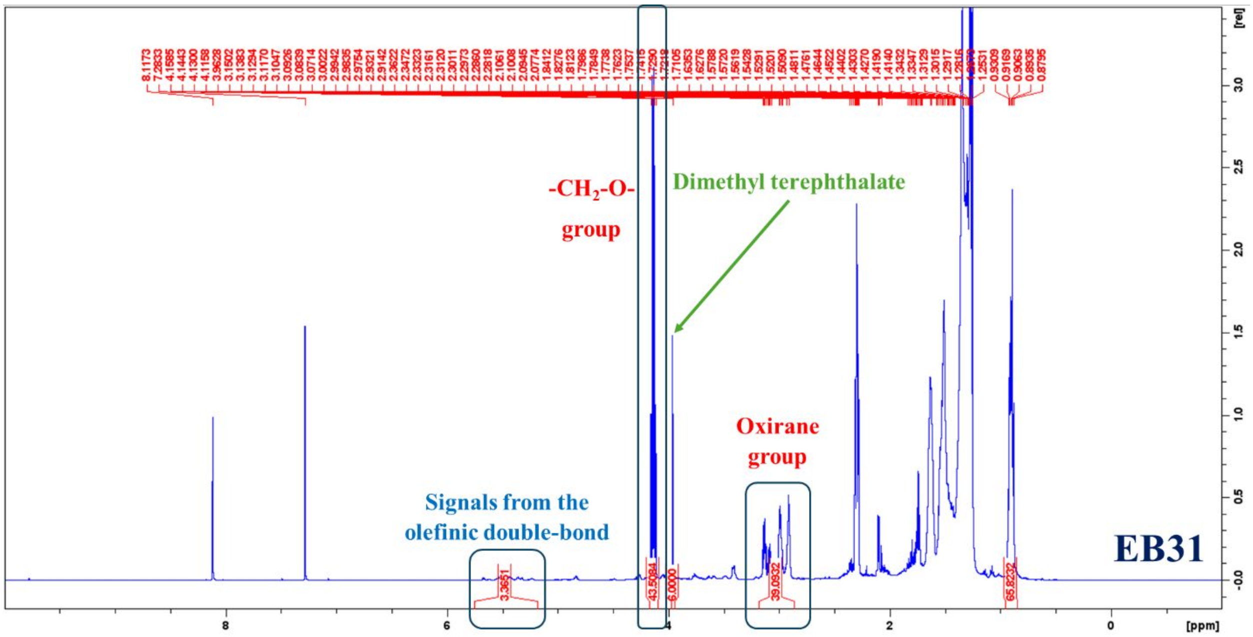

Figure S17. ^{1}H NMR (500 MHz) spectrum of the **EB31** sample (2.16% PA) in $CDCl_3$.

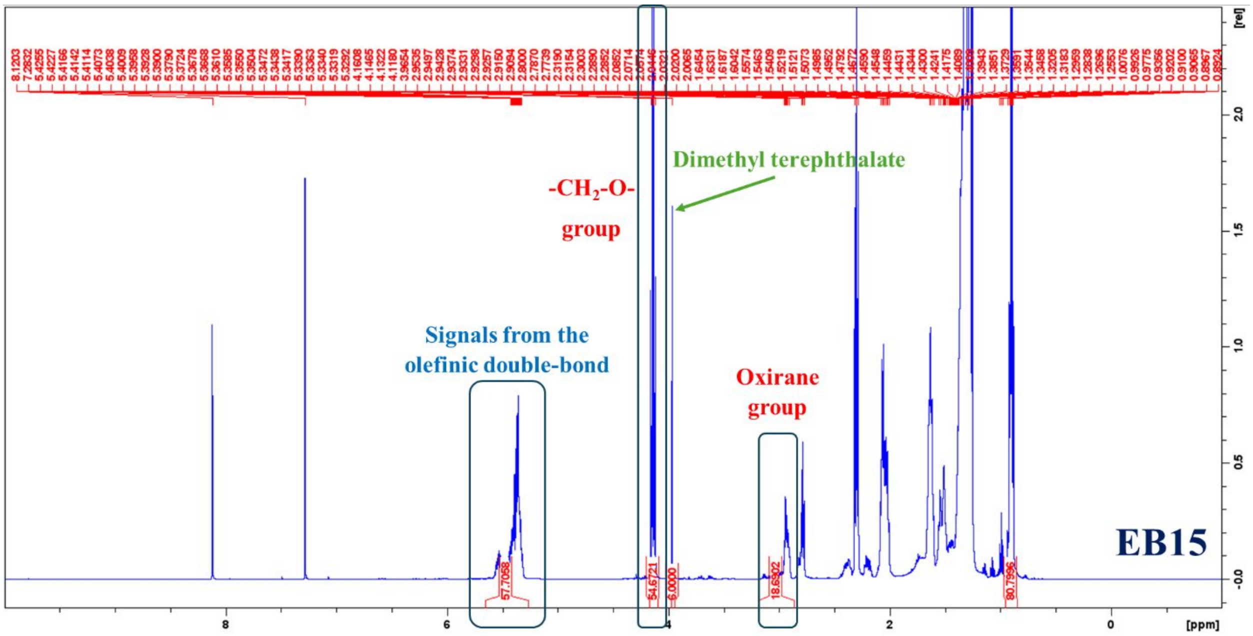

Figure S18. ^{1}H NMR (500 MHz) spectrum of the **EB15** sample (15.00% PA) in $CDCl_3$.

S5. Quantitative conversion and statistical analysis

Average Conversion of Methyl and Ethyl Biodiesel Epoxides

Table S1. Average conversion (%) of methyl and ethyl biodiesel epoxides obtained at different peracetic acid concentrations (mean ± SD, n = 3).

PAA concentration (%)	MB epoxide (sample code)	Conversion (%)	EB epoxide (sample code)	Conversion (%)
1.65	B451	64.16 ± 0.20	BE451	40.10 ± 1.16
1.67	B11	74.25 ± 0.04	BE11	76.46 ± 0.13
2.00	B32	71.83 ± 0.14	BE32	80.62 ± 0.11
2.08	B315	71.65 ± 0.19	BE315	73.32 ± 0.12
2.16	B31	59.83 ± 0.11	BE31	69.42 ± 0.13
15.00	BT	63.97 ± 0.18	BE15	27.08 ± 0.08

Statistical Analysis of Methyl Ester Epoxidation

One-way ANOVA was applied to evaluate the effect of experimental condition (PAA concentration) on the epoxidation conversion of methyl biodiesel esters.

Table S2. One-way ANOVA for epoxidation conversion of methyl biodiesel (MBE).

Source of variation	SS	df	MS	F	p-value	F critical
Between groups	495.178646	5	99.0357292	3936.8182	7.49×10^{-19}	3.10587524
Within groups	0.301875446	12	0.025156287	—	—	—
Total	495.4805214	17	—	—	—	—

Tukey's post hoc test ($p < 0.05$) was used to group conditions with statistically different mean conversions.

Table S3. Tukey HSD parameters for methyl biodiesel epoxidation (MBE).

Parameter	Value
df (within groups)	12
Number of group means (k)	6
Significance level (α)	0.05
Studentized range, q (tabulated)	4.75
MS_within (MSE)	0.025156287
Replicates per group (n)	3
Tukey HSD (DHS)	0.434966945

Table S4. Tukey post hoc grouping for mean epoxidation conversion of methyl biodiesel esters (MB) ($p < 0.05$). Different letters indicate statistically significant differences.

Sample code	PAA concentration (%)	Mean conversion (%)	SD	Tukey group
B451	1.65	64.16	0.20	c
B11	1.67	74.25	0.04	a
B32	2.00	71.83	0.14	b
B315	2.08	71.65	0.19	b
B31	2.16	59.83	0.11	d
BT	15.00	63.97	0.18	c

Statistical Analysis of Ethyl Ester Epoxidation

One-way ANOVA was applied to evaluate the effect of experimental condition (PAA concentration) on the epoxidation conversion of ethyl biodiesel esters.

Table S5. One-way ANOVA for epoxidation conversion of ethyl biodiesel (EBE).

Source of variation	SS	df	MS	F	p-value	F critical
Between groups	7399.713217	5	1479.942643	6261.022286	4.64×10^{-20}	3.105875239
Within groups	2.836487543	12	0.236373962	—	—	—
Total	7402.549705	17	—	—	—	—

Table S6. Tukey HSD parameters for ethyl biodiesel epoxidation (EBE).

Parameter	Value
df (within groups)	12
Number of group means (k)	6
Significance level (α)	0.05
Studentized range, q (tabulated)	4.75
MS_within (MSE)	0.236373962
Replicates per group (n)	3
Tukey HSD (DHS)	1.333315106

Table S7. Tukey post hoc grouping for mean epoxidation conversion of ethyl biodiesel esters (EB) ($p < 0.05$). Different letters indicate statistically significant differences.

Sample code	PAA concentration (%)	Mean conversion (%)	SD	Tukey group
BE451	1.65	40.10	1.16	e
BE11	1.67	76.46	0.13	b
BE32	2.00	80.62	0.11	a
BE315	2.08	73.32	0.12	c
BE31	2.16	69.42	0.13	d
BE15	15.00	27.08	0.08	f

S6. Integral areas used for quantitative ^{1}H NMR epoxidation analysis

This section presents the individual integrated areas obtained from quantitative ^{1}H NMR analysis, which were used to calculate the epoxidation conversion values reported in the main manuscript (Tables 8 and 9). The data are provided here to ensure analytical transparency and reproducibility, as recommended by the Reviewer.

The integrated signals correspond to the oxirane protons ($I_{epoxide}$), the terminal methyl protons ($I_{\omega-CH_3}$) used as internal reference, and the olefinic protons ($I_{C=C}$). For each experimental condition, three independent measurements were performed, and the average conversion values and associated standard deviations are reported in the main manuscript.

Table S8. Individual integral areas and calculated epoxidation conversions for methyl biodiesel esters (MB epoxides).

SAMPLE	AREA [Iepoxide]	AREA [Iω–CH3]	AREA [IC=C]	CONVERSION [%]
	37.15	72.99	5.67	
MB451	38.78	76.39	5.89	64.16 ± 0.20
	39.14	76.62	5.98	
	51.16	86.86	12.85	
MB11	48.53	82.39	12.18	74.25 ± 0.04
	46.05	78.09	11.66	
	47.56	83.46	4.04	
MB32	47.50	83.11	4.88	71.83 ± 0.14
	44.88	78.84	4.60	
	40.84	71.94	2.81	
MB315	43.68	76.54	3.04	71.65 ± 0.19
	45.45	79.96	3.13	
	34.37	72.60	2.95	
MB31	34.64	72.88	3.00	59.83 ± 0.11
	35.50	74.80	3.04	
	48.17	94.56	8.73	
MB15	43.18	85.07	7.52	63.97 ± 0.18
	40.92	80.79	7.12	

Table S9. Individual integral areas and calculated epoxidation conversions for ethyl biodiesel esters (EB epoxides).

SAMPLE	AREA [Iepoxide]	AREA [Iω–CH3]	AREA [IC=C]	CONVERSION [%]
	24.16	70.60	23.64	
EB451	27.06	78.84	26.44	40.10 ± 1.16
	22.87	70.25	22.75	
	49.37	75.44	13.50	
EB11	51.07	78.27	13.88	76.46 ± 0.13
	48.74	74.71	13.28	
	50.94	74.10	4.12	
EB32	50.30	73.10	4.09	80.62 ± 0.11
	49.24	71.43	4.00	
	42.46	67.86	13.06	
EB315	45.11	71.94	13.88	73.32 ± 0.12
	45.98	73.57	14.14	
	39.09	65.82	3.36	
EB31	40.54	68.43	3.47	69.42 ± 0.13
	39.76	67.20	3.45	
	18.69	80.79	57.70	
BE15	16.67	72.50	51.58	27.08 ± 0.08
	16.16	69.92	49.86	
